# Multiple replication origins with diverse control mechanisms in *Haloarcula hispanica*

**DOI:** 10.1093/nar/gkt1214

**Published:** 2013-11-22

**Authors:** Zhenfang Wu, Jingfang Liu, Haibo Yang, Hailong Liu, Hua Xiang

**Affiliations:** ^1^State Key Laboratory of Microbial Resources, Institute of Microbiology, Chinese Academy of Sciences, Beijing 100101, China and ^2^University of Chinese Academy of Sciences, Beijing 100049, China

## Abstract

The use of multiple replication origins in archaea is not well understood. In particular, little is known about their specific control mechanisms. Here, we investigated the active replication origins in the three replicons of a halophilic archaeon, *Haloarcula hispanica*, by extensive gene deletion, DNA mutation and genome-wide marker frequency analyses. We revealed that individual origins are specifically dependent on their co-located *cdc6* genes, and a single active origin/*cdc6* pairing is essential and sufficient for each replicon. Notably, we demonstrated that the activities of *oriC1* and *oriC2*, the two origins on the main chromosome, are differently controlled. A G-rich inverted repeat located in the internal region between the two inverted origin recognition boxes (ORBs) plays as an enhancer for *oriC1*, whereas the replication initiation at *oriC2* is negatively regulated by an ORB-rich region located downstream of *oriC2-cdc6E*, likely *via* Cdc6E-titrating. The *oriC2* placed on a plasmid is incompatible with the wild-type (but not the Δ*oriC2*) host strain, further indicating that strict control of the *oriC2* activity is important for the cell. This is the first report revealing diverse control mechanisms of origins in haloarchaea, which has provided novel insights into the use and coordination of multiple replication origins in the domain of Archaea.

## INTRODUCTION

Precise regulation of DNA replication is essential for accurate duplication of genomic information, and multiple mechanisms are involved in controlling replication initiation. DNA replication origins are genomic positions that are responsible for the initiation of DNA synthesis. DNA replication begins at a single site of origin in bacteria, whereas large numbers of replication origins are used in eukaryotes (1). As the third domain of life, archaea share similarities with both bacteria and eukaryotes, harboring bacterial-like circular chromosomes but eukaryotic-type replication apparatuses (2). The study of replication origins in several model archaea has been ongoing for more than a decade (3–14). Both unique and multiple replication origin(s) have been identified in archaeal species; as examples, a single replication origin was identified in *Pyrococcus abyssi* (3,13–14), whereas multiple origins were identified in *Sulfolobus solfataricus* and *Sulfolobus acidocaldarius* (5,6) and in the three haloarchaea, *Halobacterium* species NRC-1 (9), *Haloferax volcanii* (10) and *Haloarcula hispanica* (15). Among archaea, multiple replication origins have been best described in *Sulfolobus* species, and the studies have demonstrated three active origins in their single chromosome using 2D gel analysis and microarray-based marker frequency analysis (MFA) (5,6,16,17). Multiple replication origins were predicted to be widespread in haloarchaea (15), and they were experimentally proved in *Halobacterium* species NRC-1 (9), *H. volcanii* (10) and *H. hispanica* (15) recently.

The characterized archaeal replication origins generally possess the following important features (3–12): a region with a high content of adenine and thymine residues (AT-rich) flanked by several conserved repeats [origin recognition box (ORB)] and adjacent to a replication initiator gene, *orc1/cdc6*. These features have recently led to the *in silico* prediction of multiple replication origins for 15 haloarchaea (15). Interestingly, in contrast to the universal conservation of initiator proteins in both eukaryotes (the ORC complex) and bacteria (DnaA), a diversity of Orc1/Cdc6 homologs have been observed in archaea, especially in haloarchaea (15). A comprehensive *in silico* analysis has suggested that replication origins from haloarchaea are considerably diverse in terms of both the variant ORB elements within different origins and their adjacent *orc1/cdc6* genes (15). Although it is known that the three replication origins in *Sulfolobus* species are specifically dependent on their proximally encoded initiators (17,18), it is likely more complex in haloarchaea, as there are many more *orc1/cdc6* genes in haloarchaeal genomes than the potential replication origins (9,10,15).

Compared with the identification and characterization of replication origins, deciphering the mechanisms of the precise regulation of DNA replication initiation remains a major challenge. In bacteria, various regulatory strategies involved in controlling replication initiation have been defined [for review, see (19)]. The mechanisms are usually associated with controlling the availability and activity of the initiator protein, DnaA, at the origin, such as DnaA titration by the *datA* region (20) and DnaA autoregulation via the presence of DnaA boxes within the promoter region of the *dnaA* gene (21). In addition, other repeated sequences have been proven to be binding sites for regulatory factors and to play a crucial role in the control of replication initiation [for review, see (22)]. In recent years, more and more studies have emphasized the precise regulation of replication initiation in eukaryotes, suggesting that multiple mechanisms work to strictly control origin selection and timing of multiple replication origins [for review, see (23–25)]. In contrast, the control of replication initiation at multiple origins in archaea is far less understood. Some characterized archaeal replication origins have been shown to be directly adjacent to initiator gene (5–16), a feature reminiscent of the *oriC-dnaA* system in bacteria, thereby suggesting a bacterial-like replication-controlling mechanism in archaea. In *Sulfolobus* species, *cdc6-1* and *cdc6-3*, but not *cdc6-2*, represent strong cell-cycle-specific induction, indicating their different roles in replication control (5,26). Interestingly, serine–threonine protein kinases show cyclic induction in *Sulfolobus* species, suggesting that regulatory factors similar to eukaryotic cyclin-dependent kinase complexes may be present in archaea (26). Recently, a simple adenosine triphosphate (ATP)–adenosine diphosphate (ADP) binary switch model for Orc1/Cdc6 function has been proposed in *Sulfolobus islandicus*, which suggests that binding of ATP modulates the conformation of Orc1/Cdc6 for efficient recruitment of minichromosome maintenance helicases (MCM), whereas subsequent hydrolysis of ATP renders the Orc1/Cdc6 incapable of recruiting further MCM, thereby controlling the replication initiation (17). However, the coordination and control of the multiple and diverse replication origins in archaea remains a new area of study.

Eleven Orc1/Cdc6 homologs (hereafter denoted Cdc6) are encoded in *H. hispanica*. Seven of these homologs are predicted to be associated with replication origins, and five have been shown to possess autonomously replicating activity (15). In this study, we examined the active replication origins in the three replicons of *H. hispanica*, and determined the essential origin for each replicon. Our genetic studies further demonstrated that the multiple replication origins are specifically recognized by the initiators encoded by their adjacent *cdc6* genes. More importantly, we revealed different control mechanisms between *oriC1* and *oriC2*, the two replication origins on the main chromosome, *via* specificity of Cdc6 proteins and distinct *cis* requirements, thus providing novel insights into the diverse control mechanisms of multiple replication origins in haloarchaea.

## MATERIALS AND METHODS

### Strains, plasmids and culture

*H. hispanica* was cultivated at 37°C in AS-168 nutrient-rich medium as described previously (15), and 3 μg/ml mevinolin, 50 μg/ml uracil or 150 μg/ml 5-fluoroorotic acid (5-FOA) was added when required (27,28). For temperature experiments, cultures were grown at 28 or 42°C. For salt experiments, cultures were grown at 37°C with either 16 or 29% NaCl. The plasmids used for gene knockout and gene disruption experiments were derived from pHAR and pUBP, respectively (27,28), and pBI101 was used to identify the autonomous replication ability of replication origins (29,30). The constructed plasmids and oligonucleotides used in this study are shown in Supplementary Table S1.

### DNA and RNA preparation

Genomic DNA was purified by the phenol chloroform method. Briefly, the cell pellets were resuspended in 600 μl of double-distilled H_2_O with 100 μg/ml protease K. The samples were carefully mixed and incubated at 50°C for 30 min. The DNA was then purified by phenol chloroform extraction and ethanol precipitation. RNA was removed using RNase, and phenol chloroform extraction and ethanol precipitation were performed again. The DNA was resuspended in Tris-ethylenediaminetetraacetic acid buffer and stored at −20°C.

Total RNA was isolated using the TRIzol reagent (Invitrogen, Carlsbad, CA, USA) according to the manufacturer’s instructions. The RNA was eluted in 20 μl of RNase-free water and stored at −70°C.

### DNA microarray design

Oligonucleotide microarrays (8 × 15 K) were designed and manufactured by CapitalBio and Agilent Technologies (http://www.agilent.com), respectively, according to the whole genomic sequence of *H. hispanica* (31). Sixty-mer probes were designed for each open reading frame in the genome, and additional probes were supplied for MFA analyses to ensure a wide distribution of probes (at least one probe per kilobase) throughout the genome.

### Whole-genome DNA microarray-based MFA

MFA was performed as described (9). Genomic DNA was purified from *H. hispanica* cultures at early exponential (*OD*_600_ = 0.6) and stationary (*OD*_600_ = 2.8) phases according to the growth curve (Supplementary Figure S1). Genomic DNA (5 μg) was then labeled with Cy3-dCTP and Cy5-dCTP using 9-mer random primers (Invitrogen) and the DNA polymerase I Klenow fragment (Takara, Japan). The labeling mix contained 720-μM dATP, 720-μM dGTP and 720-μM dTTP, 120-μM dCTP and 40-μM Cy3-/Cy5-dCTP (GE Healthcare). Hybridization, washing and scanning of the arrays and data normalization were performed as described (32). Three biological replicates were performed, and the ratios (exponential versus stationary phase values) from the replicates were averaged. An MFA graph was calculated from these averaged ratios.

### Differential gene expression analysis based on DNA microarrays

The two groups of RNA samples extracted at the exponential and stationary phases were used for differential gene expression analysis based on the DNA microarrays. The experiments and data analysis were performed as described (32).

### Quantitative real-time polymerase chain reaction

Copy number determination of the regions proximal to the replication origins was performed using quantitative real-time polymerase chain reaction (PCR). *H. hispanica* and mutant strains were cultured to exponential growth phase, and the samples for real-time PCR were prepared as described (33).

Primers for real-time PCR were designed using Beacon Designer software. The SuperReal PreMix (SYBR Green) (TIANGEN, China) reagent was used for the real-time PCR, and the reaction was performed in a 20-μL volume. The real-time PCR conditions were 15 min at 95°C, 40 cycles of 10 s at 95°C for denaturation, 20 s at 60°C for annealing and 20 s at 72°C for extension. The temperature for annealing allowed for fluctuation according to the primer optimization. The real-time PCR was performed in the ‘Rotor Gene 3000’, and each experiment was repeated twice, each in triplicate.

### Gene knockout and gene disruption

Gene knockout experiments were adapted based on previously described procedures (28). Briefly, two fragments (500–800 bp in length) located immediately upstream and downstream of the target gene or origin were amplified from *H. hispanica* genomic DNA, and the products were digested with appropriate restriction enzymes and cloned into the pHAR plasmid. The resulting plasmid was then transformed into *H. hispanica* DF60 using the polyethylene glycol-mediated transformation method (34) to knock out the target gene by double-crossover homologous recombination. The mutant strains were screened using PCR analysis.

Gene disruption was performed using a single crossover method as described (27). Briefly, an ∼500-bp DNA fragment in the middle of the target gene was cloned into the pUBP plasmid. The resulting plasmid was introduced into *H. hispanica* to disrupt the target gene via single-crossover homologous recombination.

### Assay of autonomously replicating sequence activity

The autonomously replicating sequence (ARS) activity assay was performed as described previously (15). Corresponding *ori* and *ori-cdc6* deletion strains were used as transformation hosts to establish the specificity of the replication origins and *cdc6* genes. Plasmid recovery in *H. hispanica* transformants was an indicator of ARS activity and was measured by Southern blot analysis (4). For experiments assessing control mechanisms, equal amounts of plasmids (1 μg) were transformed, and the number and size of colonies and the concentration of ARS plasmids in the transformants were used as indicators of replication efficiency (35). Transformation cells were grown at 37°C for 7–8 days, the number of colonies was counted and the mean colony size was calculated with uniform colonies. For determination of the concentration of ARS plasmids in the transformants, the colonies were inoculated into 10 ml of AS-168 broth under selection, and the cells were quantified and collected at an *OD*_600_ of 2. Crude DNA in the *H. hispanica* cell lysate was fractionated by electrophoresis on 0.7% agarose gel. After transfer to a nylon membrane, hybridization-probe labeling and immunological detection were performed using DIG-High Prime DNA Labeling and the Detection Starter Kit II (Roche, USA). A DNA fragment amplified from the *bla* gene was chosen as a plasmid-specific probe.

## RESULTS

### Determination of the essential replication origins and *cdc6* genes

**Figure 1.**
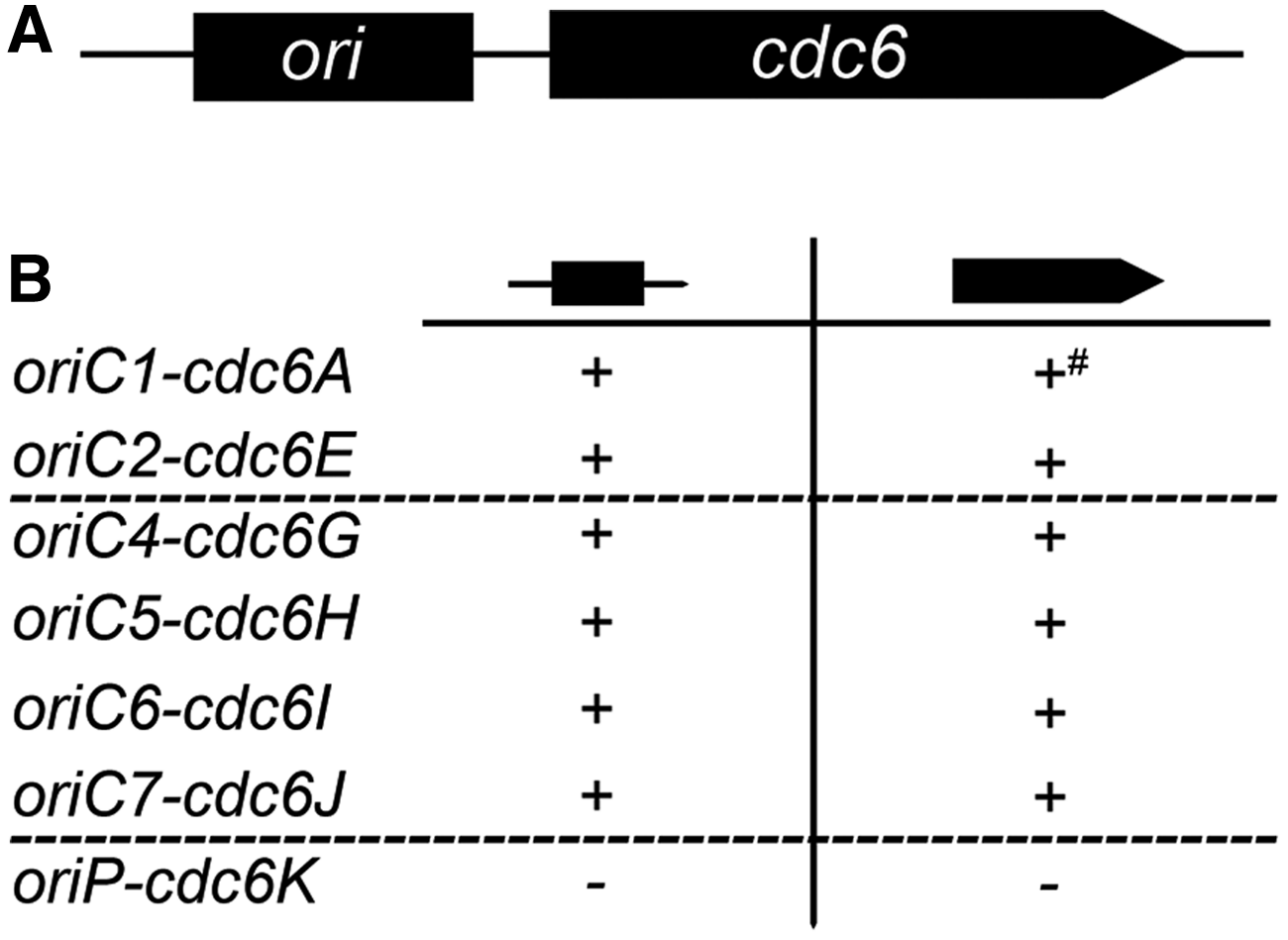
Single deletions of candidate replication origins and their adjacent *cdc6* genes. (**A**) Schematic diagram of the candidate *ori-cdc6* origin region (not to scale), including the origin region and its adjacent *cdc6* gene. (**B**) Summary of the knockout results: (+), knockouts were obtained for these targets; (−), deletions of these targets were not obtained; (+^#^), knockout of *cdc6A* gene was extremely difficult, which resulted in tiny colonies with a significant growth defect. The origins on different replicons (main chromosome, minichromosome and megaplasmid pHH400) are separated by dotted lines.

We previously predicted that there are seven replication origins in *H. hispanica*, five of which were genetically confirmed to have ARS activity (15). This raised questions about how many and which of the origins are essential for DNA replication and whether origins that are likely dormant (e.g. *oriC4-cdc6G* or *oriC5-cdc6H* on the minichromosome) will be activated when functional origins are deleted. To address these questions, the origin (*ori*) and its associated *cdc6* gene of each of the seven candidate replication origins were individually targeted for knockout (Figure 1A). Remarkably, most of the origins are dispensable in *H. hispanica*, as the two origin candidates (*oriC1* and *oriC2*) on the main chromosome and the four candidates on the minichromosome (*oriC4*, *oriC5*, *oriC6* and *oriC7*) could be knocked out, respectively, whereas only the unique origin on pHH400 (*oriP*) could not be deleted (Figure 1B and Supplementary Figure S2). Similarly, knockouts were also obtained for most of the *cdc6* genes, except *oriP-*associated *cdc6K* on pHH400 (Figure 1B and Supplementary Figure S3). Interestingly, while the *oriC1* could be readily deleted, knockout of its co-located *cdc6* gene (*cdc6A*) was extremely difficult. We did not obtain any deletants of *cdc6A* after screening >1000 Foa^r^ (5-fluoroorotic acid resistant) colonies in total (from three independent experiments), although we finally obtained one *cdc6A*-deletion strain through screening 200 additional tiny Foa^r^ colonies. This tiny-colony phenotype of the *cdc6A* knockouts is likely due to a significant growth defect of the mutants (Supplementary Figure S4). Collectively, we conclude that all of the replication origins except *oriP* (*cdc6K*) on pHH400 are dispensable, and the difficulty in knocking out the *cdc6A* gene, but not the *oriC1* region, implies a unique and vital function of Cdc6A outside of replication initiation at *oriC1*.

To investigate whether these origins can be deleted in combination, we further tried to delete the origins serially from the main and mini chromosome. Our results showed that the two active replication origins on the main chromosome, *oriC1* and *oriC2*, could not be knocked out simultaneously (Table 1 and Supplementary Figure S2). Notably, of the four candidate origins on the minichromosome, only *oriC6* and *oriC7*, which have already been proven to be functional via the ARS assay, could not be deleted simultaneously (Table 1 and Supplementary Figure S2), reinforcing the fact that these two origins are active for replication initiation on the minichromosome. Similarly, attempts to serially delete the 11 *cdc6* genes indicated that, with the exception of the *cdc6A* gene on the main chromosome, *cdc6I* or *cdc6J* on the minichromosome and *cdc6K* on pHH400, all the other *cdc6* genes could be knocked out simultaneously (Table 2). The essential *cdc6* genes were also confirmed by gene disruption experiments, where the attempts to disrupt *cdc6A* in the Δ*cdc6E* strain, *cdc6I* in the Δ*cdc6J* strain or *cdc6K* in DF60 led to no transformants or only sporadic tiny ones. Interestingly, PCR analysis of these tiny transformants, e.g. those resulted from disruption of *cdc6A* in the Δ*cdc6E* strain, revealed that they are merodiploid for the *cdc6A* gene, as both the disrupted and intact *cdc6A* genes could be observed (Supplementary Figure S5). These results further support the indispensability of these *cdc6* genes in the relevant genetic background. Altogether, our results indicate that one active *ori-cdc6* pairing on each replicon, i.e. *oriC1-cdc6A* or *oriC2-cdc6E* on the chromosome, *oriC6-cdc6I* or *oriC7-cdc6J* on the minichromosome and *oriP-cdc6K* on pHH400, is essential and sufficient for genome replication, at least under the laboratory conditions.

**Table 1.** Strains with knockouts of multiple replication origins

Attempted knockout	Knockout obtained	Replication origins present
Chromosome		
DF60Δ*oriC1*	+	*oriC2*
DF60Δ*oriC2*	+	*oriC1*
DF60Δ*oriC1ΔoriC2*	−	*oriC2*
DF60Δ*oriC2ΔoriC1*	−	*oriC1*
Minichromosome		
DF60Δ*oriC4ΔoriC5*	+	*oriC6*, *oriC7*
DF60Δ*oriC4ΔoriC5ΔoriC6*	+	*oriC7*
DF60Δ*oriC4ΔoriC5ΔoriC7*	+	*oriC6*
DF60Δ*oriC6ΔoriC7*	−	*oriC4, oriC5, oriC7*
DF60Δ*oriC7ΔoriC6*	−	*oriC4, oriC5, oriC6*

The plus (+) and minus (−) signs indicate that knockouts were obtained or not obtained, respectively.

**Table 2.** Strains with knockouts of multiple *cdc6* genes

Attempted knockout	Knockout obtained	*cdc6* genes present
Chromosome		
DF60Δ*cdc6D* Δ*cdc6E*	+	*cdc6A*, *cdc6B*, *cdc6C*, *cdc6F*
DF60Δ*cdc6D* Δ*cdc6E* Δ*cdc6C*	+	*cdc6A*, *cdc6B*, *cdc6F*
DF60Δ*cdc6D* Δ*cdc6E* Δ*cdc6C* Δ*cdc6B*	+	*cdc6A*, *cdc6F*
DF60Δ*cdc6D* Δ*cdc6E* Δ*cdc6C* Δ*cdc6B* Δ*cdc6F*	+	*cdc6A*
Minichromosome		
DF60Δ*cdc6G* Δ*cdc6H*	+	*cdc6I*, *cdc6J*
DF60Δ*cdc6G* Δ*cdc6H* Δ*cdc6I*	+	*cdc6J*
DF60Δ*cdc6G* Δ*cdc6H* Δ*cdc6J*	+	*cdc6I*
DF60Δ*cdc6I* Δ*cdc6J*	−^a^	*cdc6G*, *cdc6H*, *cdc6J*
DF60Δ*cdc6J* Δ*cdc6I*	−^a^	*cdc6G*, *cdc6H*, *cdc6I*
Genome		
DF60Δ*cdc6D* Δ*cdc6E* Δ*cdc6C* Δ*cdc6B* Δ*cdc6F* Δ*cdc6G*	+	*cdc6A*, *cdc6H*, *cdc6I*, *cdc6J*, *cdc6K*
DF60Δ*cdc6D* Δ*cdc6E* Δ*cdc6C* Δ*cdc6B* Δ*cdc6F* Δ*cdc6G* Δ*cdc6H*	+	*cdc6A*, *cdc6I*, *cdc6J*, *cdc6K*
DF60Δ*cdc6D* Δ*cdc6E* Δ*cdc6C* Δ*cdc6B* Δ*cdc6F* Δ*cdc6G* Δ*cdc6H* Δ*cdc6J*	+	*cdc6A*, *cdc6I*, *cdc6K*
DF60Δ*cdc6D* Δ*cdc6E* Δ*cdc6C* Δ*cdc6B* Δ*cdc6F* Δ*cdc6G* Δ*cdc6H* Δ*cdc6I*	+	*cdc6A*, *cdc6J*, *cdc6K*

The plus (+) and minus (−) signs indicate that knockouts were obtained or not obtained, respectively.

^a^The essentiality of either *cdc6I* or *cdc6J* was also confirmed by disruption strategy.

### Genome-wide mapping of replication origin utilization

We demonstrated that one active replication origin per replicon is essential and sufficient for *H. hispanica* genome replication. To determine whether other dormant origins are activated in the origin deletion strains, we used MFA to map the use of replication origins in *H. hispanica* wild-type and mutant strains.

**Figure 2.**
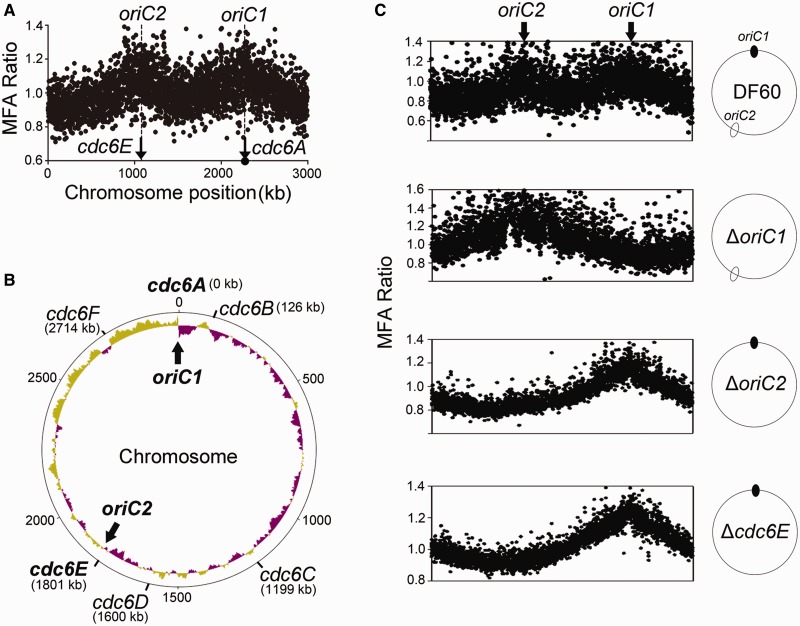
Genome-wide mapping of the chromosomal replication origins in *H. hispanica* wild-type and deletion strains. (**A**) MFA for the chromosome of the *H. hispanica* wild-type strain. The ratios of marker DNA hybridization signals of exponential to stationary phase are plotted against chromosome position (kilobase). The approximate locations of *oriC1* and *oriC2*, which are proximal to *cdc6A* and *cdc6E* (indicated with arrows), respectively, are indicated with vertical lines. (**B**) Active replication origins on the chromosome of *H. hispanica*. The start position of the chromosome is reset from the location of the haloarchaeal conserved *oriC1* origin [indicated by a round dot in (A)]. The approximate positions of the *cdc6* genes are indicated, and the active *cdc6*-associated replication origins (*oriC1* and *oriC2*) on the chromosome are in bold. The GC skew of the chromosome is represented by the inner circle. (**C**) The use of replication origins in chromosomal origin- and *cdc6*-deletion strains (Δ*oriC1*, Δ*oriC2* and Δ*cdc6E*) was monitored via MFA.

In the *H. hispanica* wild-type strain, the MFA results prominently displayed two peaks located proximal to the *cdc6A* and *cdc6E* genes on the chromosome (Figure 2A), indicating that replication of the chromosome is bidirectionally initiated from two origins, *oriC1* (*cdc6A*) and *oriC2* (*cdc6E*) (Figure 2B). Furthermore, the *cdc6A* and *cdc6E* genes on the chromosome were highly expressed in the exponential phase (Supplementary Table S2), allowing for the possibility for activation of their proximal origins, *oriC1* and *oriC2*. We then tested the replication profiles of the chromosomal *ori* deletion strains (Δ*oriC1* and Δ*oriC2*) by MFA and compared them with the replication profiles of *H. hispanica* DF60, an *H. hispanica* Δ*pyrF* strain (28) that has an identical replication profile to the *H. hispanica* WT strain (Figure 2C). Clearly, the peaks corresponding to *oriC1* and *oriC2* disappeared in the Δ*oriC1* and Δ*oriC2* strains, respectively (Figure 2C), further proving that *oriC1* (*cdc6A*) and *oriC2* (*cdc6E*) are active origins on the chromosome. Interestingly, the peak corresponding to *oriC2* also disappeared in the Δ*cdc6E* strain (Figure 2C), indicating that this origin is dependent on its adjacent *cdc6* gene, *cdc6E*. All of the MFA results were confirmed by real-time PCR (Supplementary Figure S6), and no other active origins emerged in the mutant strains. These results further confirm that a single origin, either *oriC1* (*cdc6A*) or *oriC2* (*cdc6E*), is sufficient to complete replication initiation of the *H. hispanica* chromosome.

For the remaining two small replicons, although both MFA and nucleotide disparity analyses clearly demonstrated that one origin (*oriP*) is responsible for the replication initiation of the megaplasmid, pHH400, the analyses were not able to clearly identify the active replication origins on the minichromosome (Supplementary Figure S7). Notably, of the four *cdc6* genes on the minichromosome, only *cdc6I* and *cdc6J* were highly expressed in the exponential phase (Supplementary Table S2), suggesting that their proximal origins, *oriC6* and *oriC7*, are active for replication initiation of this replicon. This is consistent with our previous results that both *oriC6-cdc6I* and *oriC7-cdc6J* show autonomous replication activities (15).

### Specific recognition and discrimination of the origin by its proximally encoded Cdc6 protein

**Figure 3.**
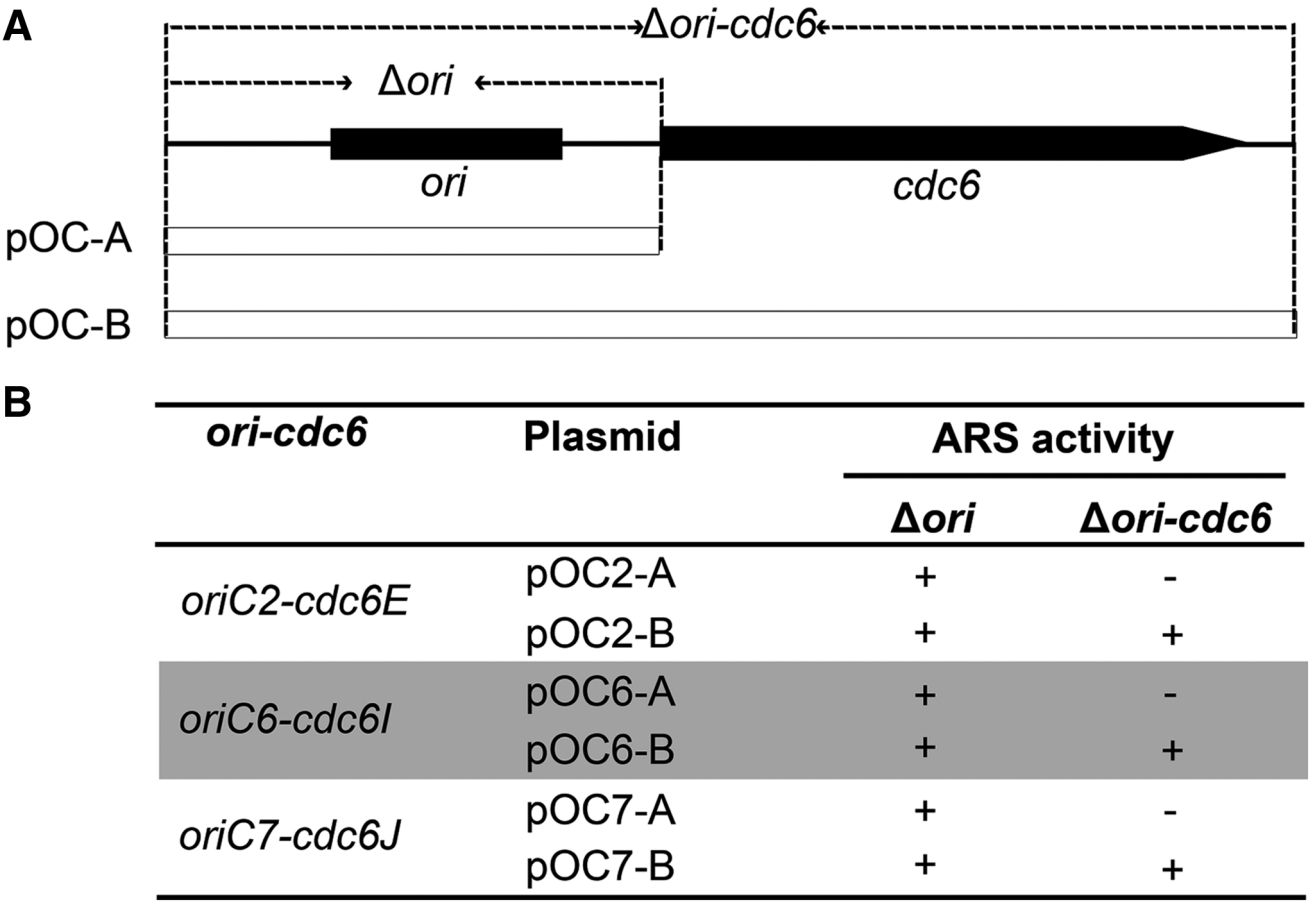
Specific recognition of replication origins by distinct Cdc6 homologs. (**A**) Schematic diagram of the *ori-cdc6* origin (not to scale) and the location of the *ori* and *ori-cdc6* deletions. Plasmids harboring an *ori* region only (pOC-A) or an *ori-cdc6* region (pOC-B) for each replication origin were constructed and subjected to ARS activity assays. Corresponding Δ*ori* and Δ*ori-cdc6* strains were used as transformation hosts. (**B**) Summary of the ARS activity assays. Plus (+) and minus (−) signs represent ARS activity and no ARS activity, respectively.

Significant consistency in the deletion results of the replication origins and *cdc6* genes (Figure 1A, Tables 1 and 2) in conjunction with the inactivity of *oriC2* in the Δ*cdc6E* strain (Figure 2C) led to the hypothesis that each origin is specified by its adjacent *cdc6* gene. To address this hypothesis, activation of these origins was investigated on a clear background where the respective *cdc6* genes were knocked out. To avoid integration, the Δ*ori* and Δ*ori-cdc6* strains were used as the transformation hosts in this assay (Figure 3A). As Δ*ori-cdc6* strains were obtained only for *oriC2-cdc6E*, *oriC6-cdc6I* and *oriC7-cdc6J* (Supplementary Figure S2), these three replication origins were designed to genetically establish the specificity of *cdc6* genes and replication origins. We first constructed the test plasmids with or without *cdc6* genes (pOC-A or pOC-B) and transformed them into the corresponding Δ*ori-cdc6* strains for ARS activity assay (Figure 3A). Briefly, pOC2-A and pOC2-B were transformed into the Δ*oriC2-cdc6E* strain, pOC6-A and pOC6-B were transformed into the Δ*oriC6-cdc6I* strain and pOC7-A and pOC7-B were transformed into the Δ*oriC7-cdc6J* strain. As summarized in Figure 3B, in all three experiments, the *ori* regions were insufficient for endowment of ARS activity in the absence of their corresponding *cdc6* genes (pOC2-A, pOC6-A and pOC7-A), whereas they obtained autonomous replication ability when the corresponding *cdc6* genes were included (pOC2-B, pOC6-B and pOC7-B). In each experiment, only the *cdc6* gene adjacent to the *ori* was deleted, indicating that each Cdc6 protein specifically recognizes its proximal origins.

To investigate whether the Cdc6 proteins are able to recognize their respective origins *in trans*, the three ARS plasmids without *cdc6* genes (pOC2-A, pOC6-A and pOC7-A) were respectively transformed into the corresponding Δ*ori* strains containing all of the *cdc6* genes (Figure 3A). Consistent with what was observed in *H. volcanii* (10), all of the three origin regions were sufficient to convey autonomous replication ability to the plasmids (Figure 3B), demonstrating that Cdc6 proteins can recognize origins *in trans* in *H. hispanica*. Together, these results clearly indicate that multiple replication origins in *H. hispanica* are discriminative and specifically recognized by their co-located Cdc6 proteins, although in principle these Cdc6 proteins can be supplied *in trans*.

### The *oriC2*-containing plasmid is incompatible with wild-type *H. hispanica*

**Figure 4.**
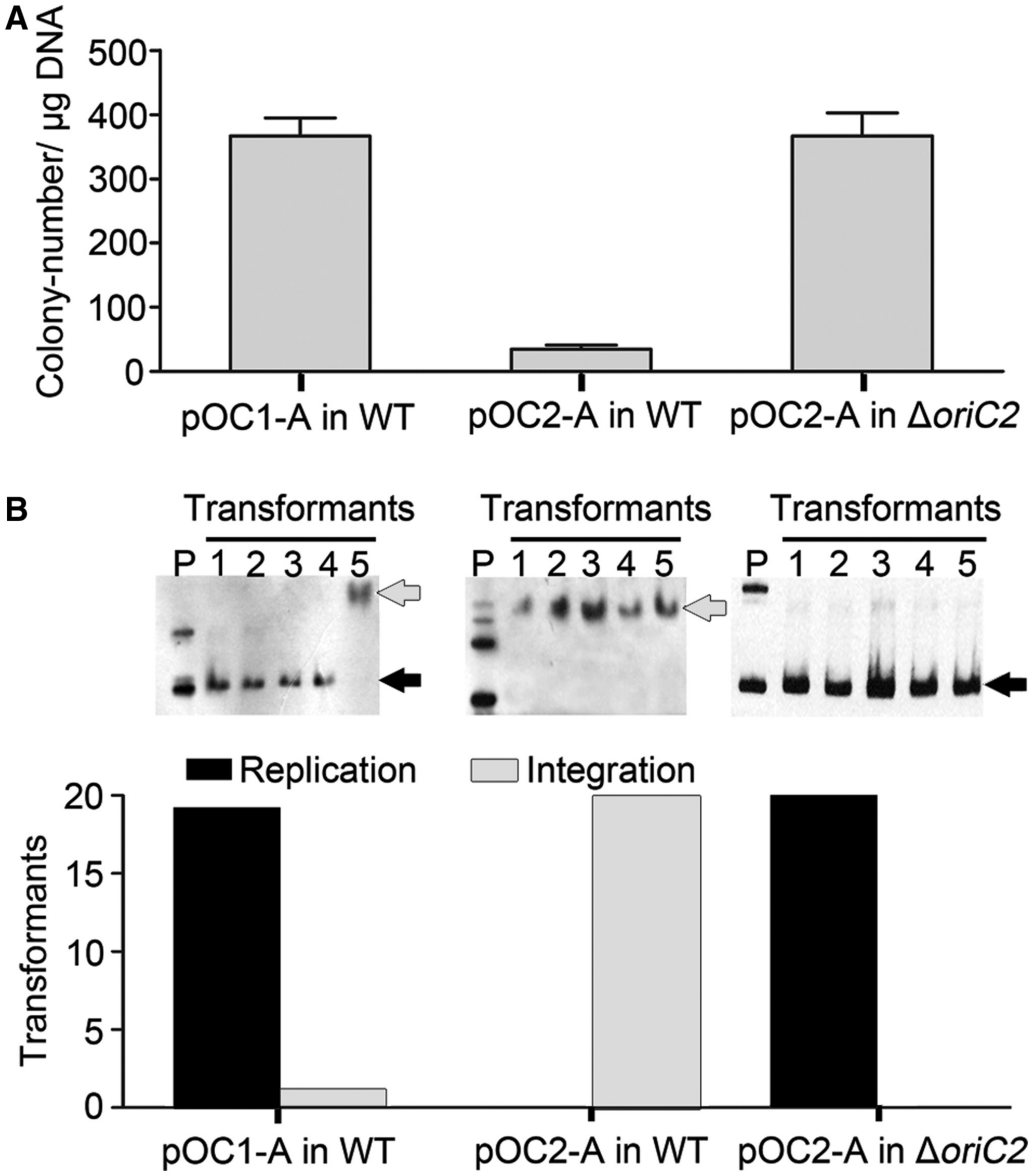
ARS plasmid containing *oriC2* is incompatible with the *H. hispanica* wild-type strain. (**A**) Transformations with the pOC1-A and pOC2-A plasmids into the *H. hispanica* wild-type strain. Transformation with pOC2-A into Δ*oriC2* strain was used as a control. Transformant number per microgram of DNA was quantified from three independent experiments. (**B**) Southern blot analysis of plasmid integration and autonomous replication led to Mev^r^ colonies, which are indicated with grey and black arrows, respectively. Lane P represents the purified plasmid, which was used as an input control. The results were quantified for 20 transformants from two independent experiments.

Because multiple *ori-cdc6* systems can coexist in one cell, we presume that distinct *ori-cdc6* systems facilitate the compatibility of multiple replication origins. To address this hypothesis, we assessed the compatibility of *oriC1*- and *oriC2*-containing plasmids with *H. hispanica*. Interestingly, compared with pOC1-A, transformation of pOC2-A into the *H. hispanica* wild-type strain led to far fewer Mev^r^ (mevinolin-resistant) colonies (Figure 4A). More interestingly, the hybridization bands corresponding to ARS plasmids were observed in the vast majority of pOC1-A transformants, whereas hybridization signals in pOC2-A transformants were observed to be associated with the high-molecular-weight genomic DNA, indicating a tendency of autonomous replication for pOC1-A but integration for pOC2-A (Figure 4B). As the length of the *oriC1* region (958 bp) is larger than that of the *oriC2* region (684 bp) in these plasmids, it is unlikely that the strong integration bias of pOC2-A is due to a larger region of homology with genomic DNA. The most likely explanation is the strict incompatibility between the *oriC2*-containing chromosome and *oriC2*-containing plasmid. To verify this speculation, we then transformed the pOC2-A into the Δ*oriC2* strain, which clearly indicated that the pOC2-A itself could replicate very efficiently in the Δ*oriC2* strain (Figure 4), as expected. This incompatibility of *oriC2* in wild-type *H. hispanica* with the *oriC2*-containing plasmid is reminiscent of origins of plasmids, whereas *oriC1* is similar to *Escherichia coli oriC*, which is not toxic to *oriC*-containing plasmid.

### A G-rich inverted repeat plays as enhancer for the haloarchaeal conserved *oriC1*

**Figure 5.**
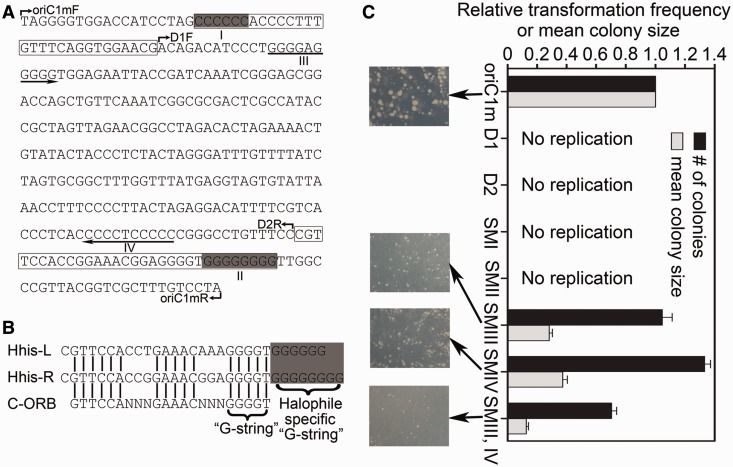
Dissection of *cis* elements for replication initiation at *oriC1*. (**A**) DNA sequence of the minimal size of *oriC1* (*oriC1m*). The ORB elements are boxed, and the extended halophile-specific ‘G-string’ elements are shaded (I and II). The G-rich inverted repeat located inside the two ORB elements is indicated with orientation arrows (III and IV). Primers for truncation analysis are indicated with bent arrows (oriC1mF and oriC1mR for *oriC1m*; D1F and oriC1mR for D1; oriC1mF and D2R for D2). (**B**) Sequence alignment of ORB elements. C-ORB represents a classic ORB element identified in archaeal origins. The ‘G-string’ and extended halophile-specific ‘G-string’ elements are indicated. (**C**) Transformations with plasmids constructed with a truncated or mutant *oriC1* origin into the Δ*oriC1* strain: *oriC1m* represents the minimal size of the *oriC1* origin; D1 and D2 denote the deletion of the left and right ORB element, respectively; SMI to SMIV denote mutants for the four G-rich sites as in (A), respectively (CCCCCC to TTTTTT in I, GGGGGGGG to AAAAAAAA in II, GGGGAGGGGG to AAAAGAAAAA in III and CCCCCTCCCC to TTTTTCTTTT in IV). Colonies were observed after 7–8 days at 37°C, and the transformation efficiency and mean colony size were quantified from three independent experiments.

To understand the difference between *oriC1* and *oriC2*, the *cis* elements of these two origins were then selected for exhaustive research. The *oriC1* origin is broadly conserved in archaea; however, when compared with the ORBs identified in *oriC1* of *S. solfataricus*, an extended halophile-specific ‘G-string’ was observed at the end of the ORBs identified in origins from haloarchaea (Figure 5A and B) (10,15), raising the question of the role of this halophile-specific motif. In addition, on sequence analysis of the *H. hispanica oriC1*, we found that apart from the inverted ORBs, each harboring a halophile-specific ‘G-string’ (I and II in Figure 5A), a perfect G-rich inverted repeat was observed directly inside the two inverted ORB elements (III and IV in Figure 5A). Based on these observations, we investigated the function of these G-rich motifs for origin initiation at *oriC1* of *H. hispanica*.

We first identified the minimal size of *oriC1* for promoting efficient autonomous replication, including an AT-rich region flanked by two inverted ORB elements (*oriC1m* in Figure 5A). Deletion of any one of the two ORB elements impaired the autonomous replication ability of *oriC1m* (D1 and D2 in Figure 5C). To determine the roles of the two G-rich repeats identified in *oriC1*, we designed mutations in the repeats (SMI, SMII, SMIII and SMIV). Interestingly, variation in any one of the two ‘G-strings’ at the end of the ORBs (SMI or SMII) led to the loss of functionality of *oriC1m* (Figure 5C), indicating that the halophile-specific ‘G-string’ elements within ORBs are essential for *oriC1*. Furthermore, we found that mutation of the G-rich inverted repeat inside the ORB elements (SMIII and SMIV) led to smaller colony sizes, especially on simultaneous mutation of both sides of the inverted repeat (SMIII, IV) (Figure 5C), suggesting that the G-rich inverted repeat, although not an essential part of *oriC1*, significantly promotes the initiation efficiency of *oriC1*. Thus, the G-rich inverted repeat plays an important role as an enhancer for replication initiation at *oriC1*. Interestingly, this G-rich inverted repeat was observed at *oriC1* origins from most haloarchaea (Supplementary Figure S8), suggesting that the enhancer activity of this repeat for *oriC1* is pervasive in haloarchaea. In addition, only half of the G-rich inverted repeat was observed in *Halobacterium* species NRC-1, *Halogeometricum borinquense*, *Halomicrobium mukohataei* and *Halorhabdus utahensis* (Supplementary Figure S8). In consideration of the differential origin efficiencies observed when the G-rich inverted repeat of *H. hispanica oriC1* was altered (Figure 5C), the data strongly suggest that the distribution of this repeat controls the origin efficiency of *oriC1* in different haloarchaea.

### Negative control of *oriC2* by a separated ORB-rich region

**Figure 6.**
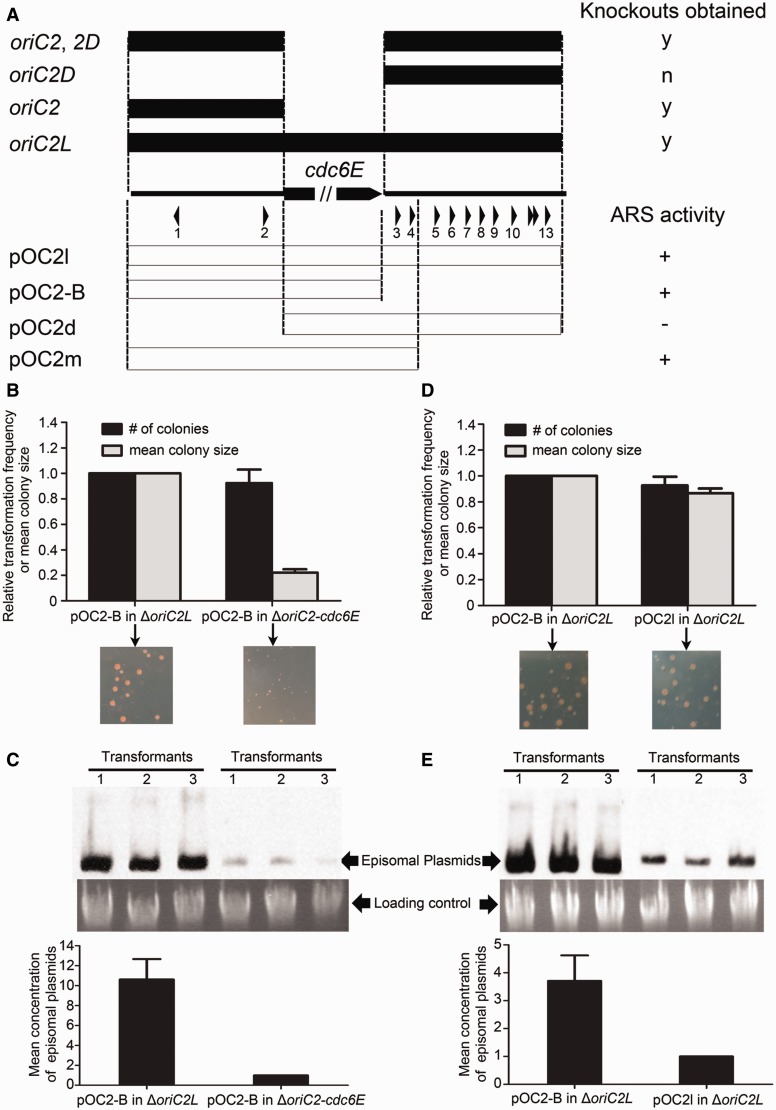
Negative control of replication origin activity at *oriC2*. (**A**) Knockout analyses and ARS assays of the *oriC2* region. The ORB elements found in the intergenic regions flanking the *cdc6E* gene are indicated with numbered arrowheads. The yes (y) and no (n) signs in the knockout analyses indicate that knockouts were obtained (y) or not obtained (n). The plus (+) and minus (−) signs indicate ARS activity and no ARS activity, respectively. (**B**, **D**) Transformations with pOC2-B into Δ*oriC2L* and Δ*oriC2-cdc6E* (B), and pOC2-B and pOC2l into Δ*oriC2L* (D). Transformants were observed after 7–8 days at 37°C, and the transformation efficiency and mean colony size were quantified from three independent experiments. (**C**, **E**) Southern blot analysis of ARS plasmids in transformants as in (B) and (D) using a *bla* gene probe. The cells were quantified via OD measurements, and genomic DNA was used as a loading control. For each transformation, three colonies were repeated for Southern blot analysis, and the mean concentration of episomal plasmids was quantified from three independent experiments.

For the other active origin on the main chromosome, *oriC2*, we surprisingly found that, apart from the *oriC2* locus located upstream of the *cdc6E* gene, there is an additional cluster of Cdc6 binding elements (11 ORB elements in the 722-bp intergenic region, named *oriC2D* for simplicity) located directly downstream of the *cdc6E* gene (Figure 6A). This distinct structure of *H. hispanica oriC2* suggests that it may use a different replication modulation strategy from *oriC1*. To address this question, we conducted genetic experiments to determine the role of the *oriC2D* region.

Interestingly, whereas the *oriC2* region could be readily knocked out from the genome (Figures 1 and 6A)*,* knockout of *oriC2D* was unsuccessful (Figure 6A and Supplementary Figure S9). However, the *oriC2D* region could be readily deleted when simultaneous deletion of *oriC2-cdc6E* was performed (Δ*oriC2L*) or when the *oriC2* deletion strain was used (Δ*oriC2*, *2D*) (Figure 6A and Supplementary Figure S9). These data strongly suggest an important role of *oriC2D* in the control of replication initiation at *oriC2*.

In the ARS activity assay, it was shown that the *oriC2D* region alone was insufficient to drive autonomous replication (pOC2d in Figure 6A). To test how this *oriC2D* region affects the ARS activity of *oriC2*, we first tested the ARS activity of pOC2-B in the strains with or without the *oriC2D* region (Δ*oriC2-cdc6E* or Δ*oriC2L*). The Δ*oriC2-cdc6E* strain transformed with pOC2-B grew to a significantly smaller colony size (Figure 6B), and the concentration of pOC2-B in the transformants was extremely low (Figure 6C), indicating a lower replication efficiency of pOC2-B in Δ*oriC2-cdc6E* than in Δ*oriC2L*. These results indicate that the *oriC2D* region on the chromosome in the Δ*oriC2-cdc6E* strain reduces the ARS activity of pOC2-B. Furthermore, we constructed the ARS plasmid with the *oriC2D* region (pOC2l in Figure 6A) and tested its ARS activity in the Δ*oriC2L* strain. Although there was no significant difference between the colony sizes of the transformants with pOC2-B and pOC2l (Figure 6D), Southern blot analysis revealed that the concentration of pOC2-B in the transformants was much higher than that of pOC2l (Figure 6E), indicating that pOC2-B replicates with higher efficiency than pOC2l. Together, our results suggest that *oriC2D* does not exhibit origin activity but appears to play an essential role in negative regulation of the activity of *oriC2*. In consideration of the abundance of ORB elements in the *oriC2D* region, it is likely that *oriC2D* has a high affinity for the Cdc6E protein and hence adjusts the concentration and activity of Cdc6E at *oriC2*, thereby accurately modulating replication initiation efficiency at *oriC2*. The lethality of Δ*oriC2D* may be due to an over-initiation of replication at *oriC2*. These results and previous observation of incompatibility of *oriC2*-containing plasmid with the wild-type (but not the Δ*oriC2*) strain indicate that strict control of the *oriC2* activity is important for the cell. It is noteworthy that deletion of *oriC1* did not change the requirement of the negative regulation of replication initiation at *oriC2*, as knockout of *oriC2D* was also not successful in the Δ*oriC1* strain (Supplementary Figure S9). These results also indicate that the *oriC1* and *oriC2* are controlled independently.

The *oriC2* homolog was found in *Halorubrum lacusprofundi*, but only three ORB elements were observed in its *oriC2D* region, indicating that the number of ORB elements may control the extent of *oriC2D* function (Supplementary Figure S10). To address this hypothesis, we constructed a plasmid with a fragment containing the *oriC2-cdc6E* and two downstream ORB elements (pOC2m in Figure 6A) and tested its ARS activity in the Δ*oriC2L* strain. As expected, pOC2m showed a moderate concentration between pOC2-B and pOC2l (Supplementary Figure S10), reinforcing the mechanism in which the distribution and number of ORB elements control origin efficiency via a Cdc6E-titrating activity. More interestingly, ORB elements were also observed downstream of many other predicted *ori-cdc6* regions in haloarchaea (15), suggesting that this negative control strategy of replication origin is widely used by haloarchaea.

### Growth patterns of *oriC1* and *oriC2* deletion strains under different conditions

**Figure 7.**
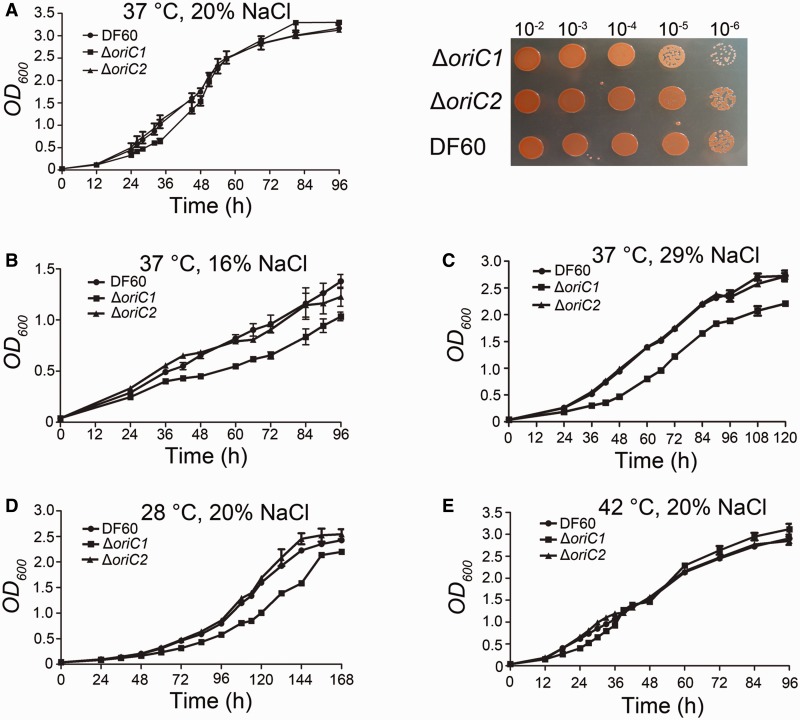
Growth curves of chromosomal origin deletion strains determined under different conditions. *H. hispanica* cultures were grown under standard conditions (37°C, 20% NaCl) (**A**): (left) growth at 37°C with shaking at 200 rpm; (right) Equal amounts of serial dilutions of exponentially growing cells (*OD_600_* = ∼1.5) were spotted on AS-168 (20% NaCl) supplemented with uracil and grown for 7 days at 37°C. For salinity, the lowest salt concentration (16% NaCl) in which *H. hispanica* can survive (**B**) and a saturated salt concentration (**C**) were chosen. For temperature conditions, cultures were grown in nutrient-rich AS-168 medium at low (**D**) and high (**E**) temperatures.

As the *oriC1* and *oriC2* are quite different but located on the same chromosome, it is interesting to investigate their individual role under different culture conditions. To address this question, we determined growth patterns of *oriC1* and *oriC2* deletion strains under different conditions, including normal experimental conditions (20% NaCl and 37°C), high and low salinity (29% and 16% NaCl, respectively) and two temperatures (42°C and 28°C). Under normal conditions, the *oriC1* deletion strain showed slightly slow growth at the early exponential phase, whereas the *oriC2* deletion strain showed no obvious growth defects (Figure 7A). Interestingly, the growth profiles of Δ*oriC2* under all detected conditions were indistinguishable from the DF60 strain (wild-type), but Δ*oriC1* showed growth defects in many different conditions (Figure 7B–E). Remarkably, under both high and low salinities as well as low temperature, all of which are slow-growing conditions for *H. hispanica*, growth defects of the Δ*oriC1* strain were more obvious than those observed under normal conditions (Figure 7B–D); these growth defects were rescued under fast-growing conditions (high temperature) (Figure 7E). Thus, these data suggest that the two origins on the chromosome may be differently used, and *oriC1* exhibits a broader adaptation.

## DISCUSSION

DNA replication initiation and its precise regulation depend on the interaction of *trans*-acting factors (initiators and regulators) with *cis*-acting sequence elements. Diverse mechanisms have evolved to ensure the precise regulation of replication initiation events, and these mechanisms have been well characterized in bacteria and unicellular eukaryotes. In contrast, a focus on archaeal replication origins has only begun relatively recently, and little is known about origin utilization and control in archaea. Among archaea, three replication origins in the single chromosome of *Sulfolobus* species have been known for about a decade (5–7,16–18). Bioinformatics analyses have predicted multiple replication origins in most haloarchaea (15), and this prediction was experimentally demonstrated in *Halobacterium* species NRC-1 (9), *H. volcanii* (10) and *H. hispanica* (15); however, due to the complex multireplicon structure, deciphering origin utilization across the haloarchaeal genomes remains difficult. In addition, the mechanism of replication control in archaea is still unclear, and again the multireplicon structure of haloarchaeal genomes makes an understanding of the control and coordination of replication initiation at multiple origins more difficult and intriguing. In this report, we not only demonstrate the use of distinct *ori-cdc6* origins on the three replicons of *H. hispanica* but also provide the first evidence of distinct control strategies used by different origins in archaea.

We first combined knockout and MFA approaches to determine the essential origins for the three replicons of *H. hispanica*, indicating that one active replication origin per replicon was essential and sufficient for genome replication and cell viability. A comparative genomic analysis of extrachromosomal replicons of *H. hispanica* and *Haloarcula marismortui* has revealed that multiple replication origins may be important for construction and/or rearrangement of minireplicons (15). Thus, it is easy to conclude that multiple replication origins on different replicons play an important role in generating novel replicons and maintaining the multireplicon structure of haloarchaeal genomes. We then characterized the two replication origins on the chromosome to determine the basis for the presence of multiple origins per replicon. Our data suggest a model whereby *oriC2-cdc6E* was integrated into an ancestral chromosome that was dependent on *oriC1-cdc6A*; this model is supported by the fact that the plasmid containing *oriC2*, but not *oriC1*, is incompatible with *H. hispanica*, that replication initiation at *oriC2* is negatively controlled by an additional ORB-rich region (*oriC2D*) and that *oriC1* exhibits better adaptation to common environmental conditions. We speculate that acquisition of *oriC2* was accompanied by capture of new genomic content, similar to the observations from origin comparison between *Aeropyrum pernix* and *Sulfolobus* species (7) and the hypothesis of gene displacement by homologs from plasmids and viruses (36). Together, multiple replication origins may account for the increase in genome size, and the inactive origins may be due to incomplete acquisition or inactivation under normal replication conditions.

It is still not clear why a large number of *cdc6* genes are present in most species of haloarchaea (37). Deletion analysis of *cdc6* genes in *Halobacterium* species NRC-1 (38) and *H. volcanii* (10) (denoted *orc* genes in these two species) suggested the possibility of functional overlap among the multiple Cdc6 homologs in haloarchaea. *H. hispanica* encodes 11 *cdc6* genes, of which seven were predicted to associate with origin candidates (15). Here, we constructed the minimal strains containing *cdc6A* on the chromosome, *cdc6I* or *cdc6J* on the minichromosome and *cdc6K* on pHH400, and these strains were highly consistent with the deletion analysis of replication origins. In addition, our comprehensive genetic analysis revealed that the ARS activity of each origin is specifically dependent on its adjacent *cdc6* gene (Figure 3), in accordance with the MFA result demonstrating that the peak corresponding to *oriC2* disappeared in the Δ*cdc6E* strain (Figure 2C). Thus, although the analysis does not include the *cdc6* genes without adjacent origins, our data suggest that the large number of *cdc6* homologs, at least in part, plays an important role in specifying distinct replication origins in *H. hispanica*. This specification contributes to distinct *ori-cdc6* systems, which may have at least three evolutionary advantages; one is ensuring the compatibility of multiple replication origins and therefore multiple replicons, and this strategy accounts for the fact that the *oriC2*-containing plasmid is incompatible with *H. hispanica*. Two more evolutionary advantages of having distinct *ori-cdc6* systems are minimizing competition among multiple origins for initiators and maintaining independent control of replication initiation at different origins. Importantly, requiring distinct initiators for the origins on different replicons may be favorable for replicon-specific replication control, such as the copy number of multireplicons during growth phases (33) and the up- or downregulation of genes on one of the replicons (39), under certain environmental conditions. In addition, although initiator proteins have been demonstrated to be strictly *cis*-acting in several cases (4,40,41), the *cis* location of the *cdc6* gene and origin is not required for the ARS activity in *H. hispanica*. The reason for this may be the divergent Cdc6 proteins and their specific recognition of ORB elements within different origins. Direct linkage of the *cdc6* gene to the origin may facilitate its transcription directly after replication initiation to sequentially control its cognate origin, similar to the bacterial *oriC-dnaA* system and reminiscent of bacterial-like control mechanisms.

Replication initiation in *E. coli* is highly regulated, and multiple mechanisms have been defined (19). Interestingly, our results suggested that some similar mechanisms may be used by different replication origins in *H. hispanica*. A perfect G-rich inverted repeat directly inside each ORB element of *H. hispanica oriC1* was shown to be a replication enhancer, which stimulated origin activation at *oriC1* (Figure 5). In contrast, an abundance of ORB elements (*oriC2D*) was observed directly downstream of the *oriC2-cdc6E* region and played an essential role in negatively controlling origin efficiency at *oriC2* (Figure 6). Various repeated sequences function to control replication initiation by initiator protein or regulatory factor binding in *E. coli* (22). Similarly, because of its close location to ORB elements, we propose that the G-rich inverted repeat assists the binding activity of initiator or regulatory factors at *oriC1* and therefore confers replication enhancer activity. The negative control of replication initiation due to DnaA-titrating activity at the *datA* locus has been described in *E. coli* (20). Similarly, our experimental data in conjunction with the high density of ORB elements suggest that the *oriC2D* region negatively controls *oriC2* activity, likely due to its high binding capacity of the Cdc6E protein, which effectively regulates the activity of Cdc6E at *oriC2*. More interestingly, our analysis of other haloarchaeal replication origins also suggests that the regulatory strategies based on *cis* elements are widely used in haloarchaea (15).

Although distinct *ori-cdc6* origins and their different control mechanisms were observed, we presume that there are common *cis* elements and factors for the use and coordination of the multiple replication origins because they coexist in one cell. Distinct from the ORBs identified in *oriC1* of *S. solfataricus* (5), an extended halophile-specific ‘G-string’ element has been identified at the end of each ORB in haloarchaea (15). Here, based on *oriC1* of *H. hispanica*, we found that these halophile-specific motifs are essential for the autonomous replication of *oriC1*. A characteristic ‘G-string’ element observed in many archaeal ORBs has been shown to be recognized by the AAA+ domain of the Orc1/Cdc6 protein (42,43). Thus, it is likely that the extended halophile-specific G-stretches in haloarchaeal ORBs may play an essential role in the interaction between the Orc1/Cdc6 protein and its origin at high intracellular salt concentrations. The two chromosomes of *Vibrio cholerae* use distinct mechanisms for their replication control, but share some common factors including DnaA and DNA methylation to ensure the coordination of their replication (44,45). The extreme difficulty in knocking out the *cdc6A* gene, but not its associated *oriC1* region, suggests a vital function of the Cdc6A protein outside of replication initiation at *oriC1*. Similar to the *V. cholerae* DnaA, the Cdc6A protein may function as a common factor for coordination of replication initiation at multiple origins in *H. hispanica*. Recently, work on replication initiators from both bacteria and eukaryotes has demonstrated that they participate in diverse processes besides their well-known activity in DNA replication, including chromosome segregation, cell division and cell cycle regulation (46). It is likely that Cdc6A has a similar role in the transcriptional regulation of cellular processes outside of replication initiation to coordinate replication initiation of the three replicons with the cell cycle in *H. hispanica*. Furthermore, as all the five active replication origins are dependent on Orc1/Cdc6 proteins, it is possible to coordinate replication initiation at these origins by conformational changes of Orc1/Cdc6 proteins via an ATP–ADP binary switch, which has been recently proposed in *S. islandicus* (17). In this regard, our results indicate that *H. hispanica* offers great potential for a more detailed understanding of the control and coordination of multiple replication origins in haloarchaeal multireplicon genomes.

## SUPPLEMENTARY DATA

Supplementary Data are available at NAR Online.

## FUNDING

This work was partially supported by grants from the National Natural Science Foundation of China [30925001, 31100893, 31271334] and the Chinese Academy of Sciences [KSCX2-EW-G-2-4]. Funding for open access charge: National Natural Science Foundation of China [30925001].

*Conflict of interest statement*. None declared.

## Supplementary Material

Supplementary Data
