# Supplementary material for: Multiple replication origins with diverse control mechanisms in Haloarcula hispanica
Source: Nucleic Acids Res. 2013 Nov 22;42(4):2282–94. doi: 10.1093/nar/gkt1214 (PMC3936714; doi:10.1093/nar/gkt1214)
Supplement: Supplementary Data [file supp_42_4_2282__index.html]

Multiple replication origins with diverse control mechanisms in Haloarcula hispanica — Multiple replication origins with diverse control mechanisms in Haloarcula hispanica — Supplementary Data 

# Multiple replication origins with diverse control mechanisms in *Haloarcula hispanica*

## Supplementary Data

files

**Files in this Data Supplement:**

- Supplementary Data - pdf file
