# Supplementary material for: Multiple replication origins with diverse control mechanisms in Haloarcula hispanica
Source: Nucleic Acids Res. 2013 Nov 22;42(4):2282–94. doi: 10.1093/nar/gkt1214 (PMC3936714; doi:10.1093/nar/gkt1214)
Supplement: Supplementary Data [file supp_gkt1214_nar-02070-v-2013-File009.pdf]

Supplementary information

Multiple replication origins with diverse control mechanisms in *Haloarcula hispanica*

Zhenfang Wu[1,2], Jingfang Liu[1], Haibo Yang[1,2], Hailong Liu[1], Jian Zhou[1], Hua Xiang[1,*]

[1] *State Key Laboratory of Microbial Resources, Institute of Microbiology, Chinese Academy of Sciences, Beijing, 100101, China;* [2]*University of Chinese Academy of Sciences, Beijing, China*

* To whom correspondence should be addressed. Tel: 86-10-6480-7472; Fax: 86-10-6480-7472; Email: xiangh@ im.ac.cn

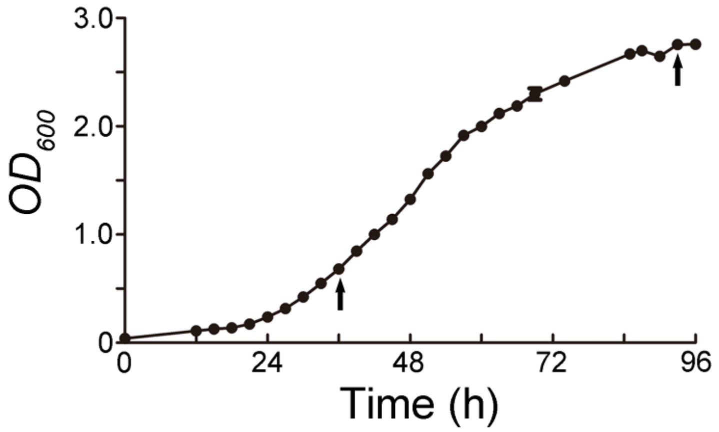

Supplementary Figure S1. Growth curve of *H. hispanica*. The time points at which cultures were harvested for marker frequency are indicated with arrows.

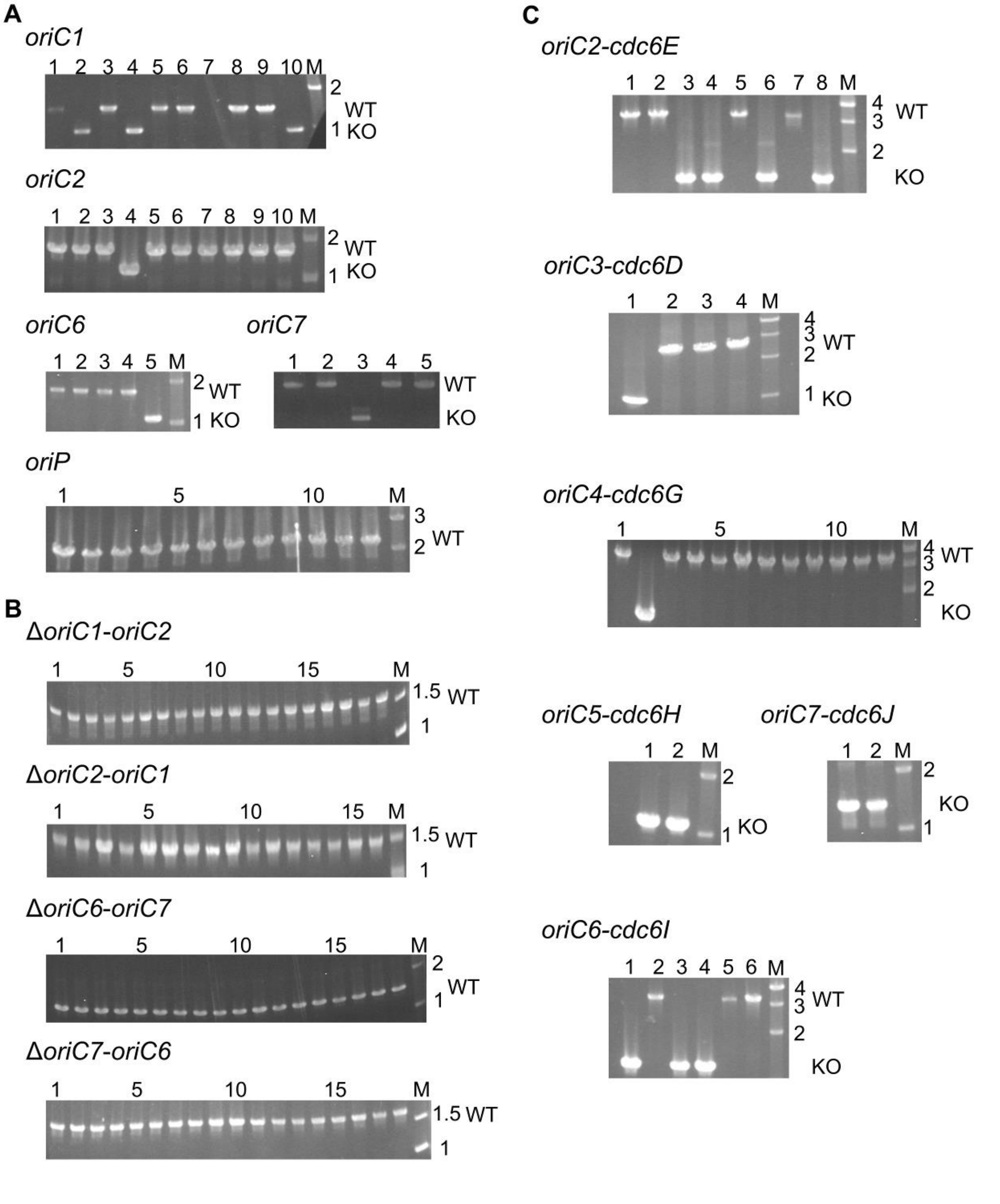

Supplementary Figure S2. PCR assay to screen for knockouts of *H. hispanica* replication origins. Crude DNA from individual Foa^r colonies was used as template, and forward and backward primers were located ~500 bp up- and down-stream of each target, respectively. Lane M denotes DNA ladder. WT and KO respectively denote wild-type and knockout alleles. The target was considered to be essential after more than 200 Foa^r colonies (including tiny colonies if available) were screened without recovering a single knockout in at least two independent experiments. (A) Knockout assays for origin region (*ori*). (B) Simultaneous-knockout assays for active replication origins on chromosome or minichromosome. (C) PCR screening for knockouts of *ori-cdc6* regions for transformation hosts.

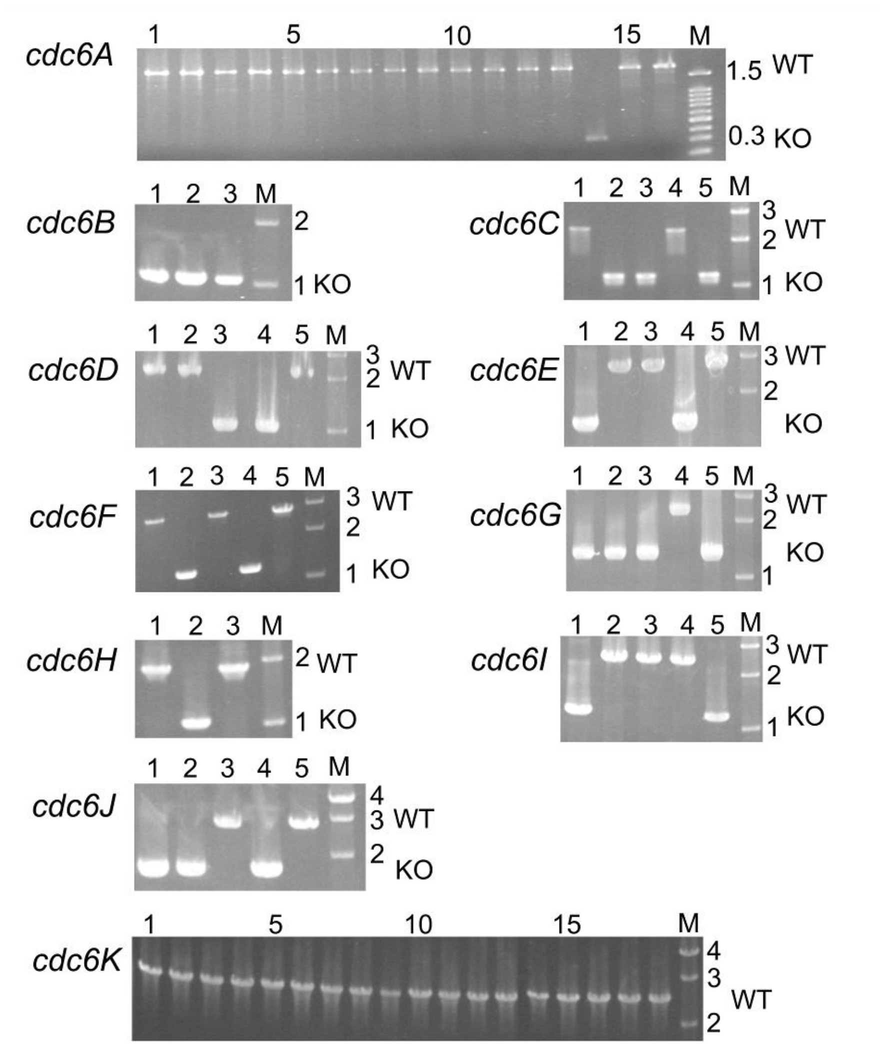

Supplementary Figure S3. PCR assay to screen for knockouts of *H. hispanica cdc6* genes. Crude DNA from individual Foa^r colonies was used as template, and forward and backward primers were located ~500 bp up- and down-stream of each *cdc6* gene, respectively (~250 bp upstream and ~80 bp downstream of *cdc6A*). Lane M denotes DNA ladder. WT and KO respectively denote wild-type and knockout alleles. After screening more than 1000 Foa^r colonies without recovering a single knockout in three independent experiments, we obtained one *cdc6A*-deletion strain via screening more than 200 tiny colonies. For *cdc6K* gene, more than 500 Foa^r colonies (including tiny colonies if available) were screened without recovering a single knockout in three independent experiments.

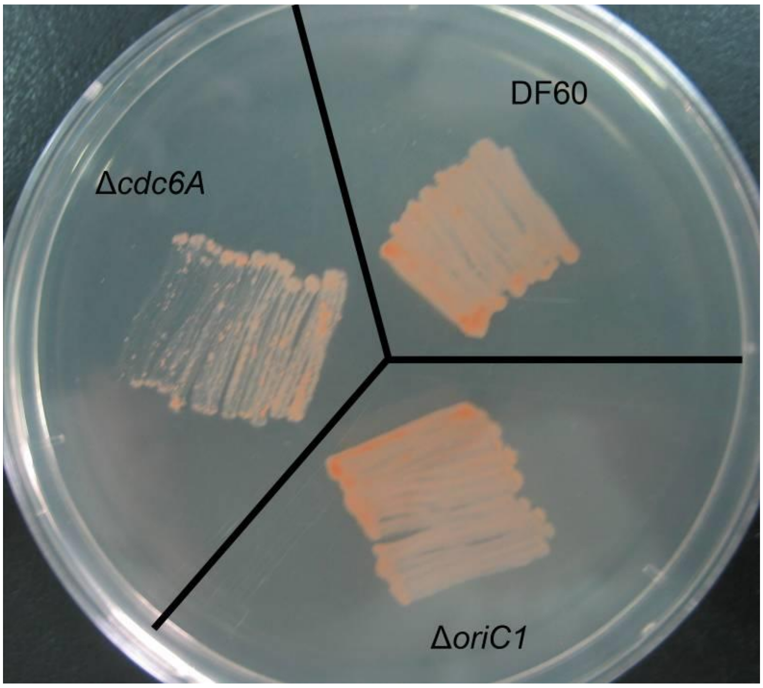

Supplementary Figure S4. Growth defect of the *cdc6A*-deletion strain. DF60, Δ*oriC1* and Δ*cdc6A* were grown on AS-168 plate supplemented with uracil for 6 d at 37 ℃. The results indicated that the *cdc6A*- but not *oriC1*-deletion strain displayed a significant growth defect.

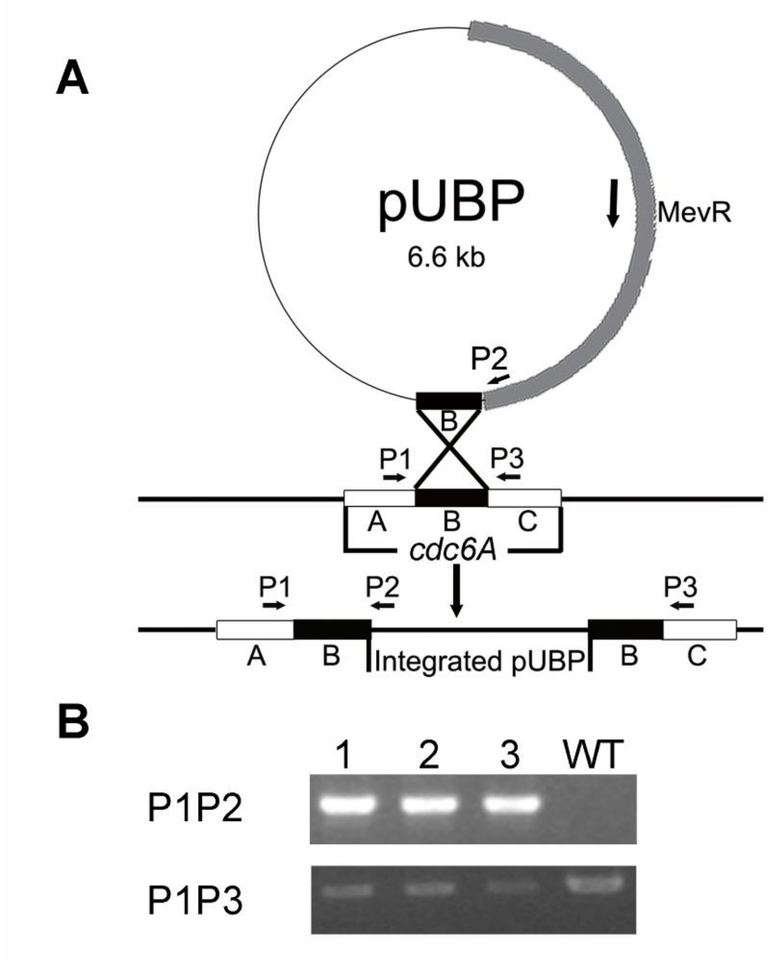

Supplementary Figure S5. PCR analysis of attempted disruption of *cdc6A* gene in Δ*cdc6E* strain. (A) Schematic representation of disruption of *cdc6A* gene via a single-crossover strategy. Primers P1 and P2 were used to confirm integration of pUBP at the *cdc6A* locus, and P1 and P3 were used to determine presence or absence of an intact *cdc6A* gene. (B) PCR analysis of three of the tiny colonies isolated on selective medium. All three still contain an intact *cdc6A* gene (P1P3), suggesting that the *cdc6A* gene cannot be completely disrupted in Δ*cdc6E* strain.

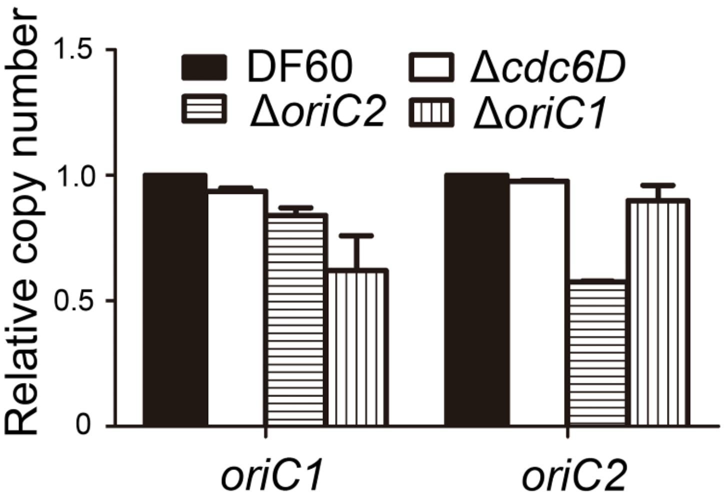

Supplementary Figure S6. Quantitative real-time PCR confirmation of the MFA results. Copy number of the regions proximal to *oriC1* and *oriC2* was determined (Supplementary Table 1 for primers). The results indicated that copy number of the regions proximal to *oriC1* and *oriC2* respectively decreased in Δ*oriC1* and Δ*oriC2* strains.

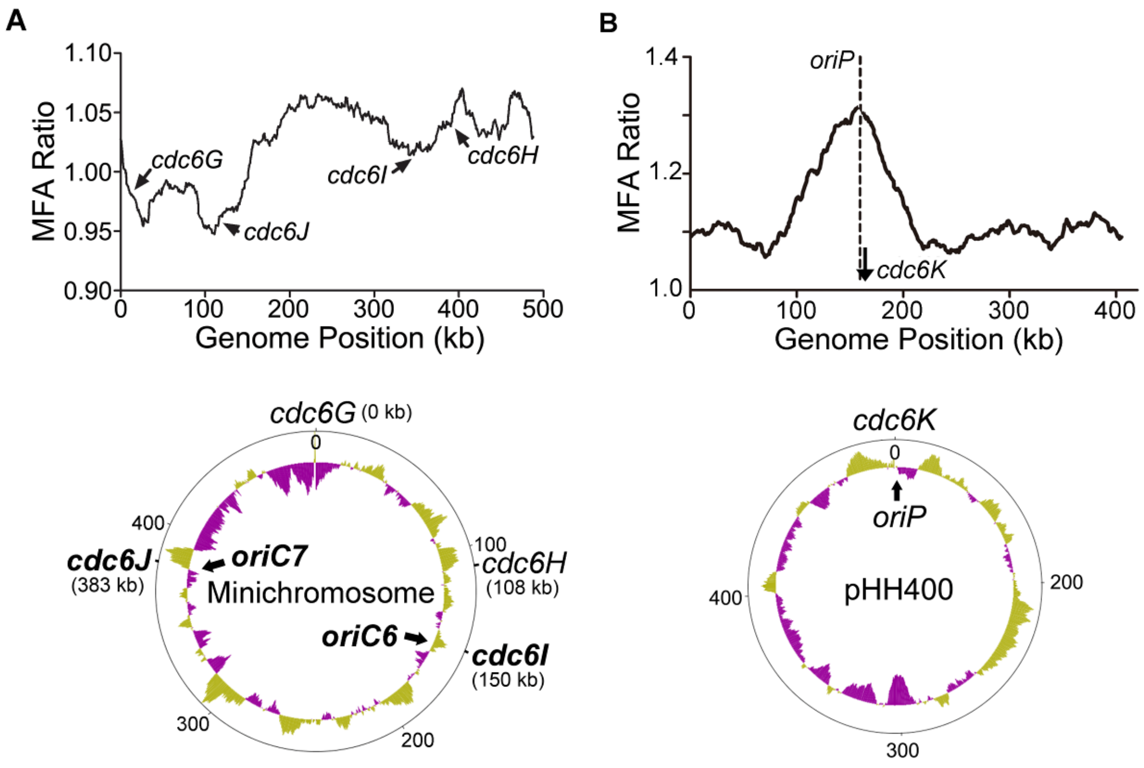

Supplementary Figure S7. Genome-wide mapping of replication origins on extrachromosomal replicons of *H. hispanica*. (A) (Up) MFA for minichromosome of *H .hispanica*. (Down) the replication origins on the minichromosome. The functional origins (*oriC6* and *oriC7*) indentified via ARS assays are bolded. The approximate positions of the *cdc6* genes are indicated, and the *cdc6* genes that are up-regulated in the exponential phase (*cdc6I* and *cdc6J*) are bolded. (B) MFA for pHH400 of *H. hispanica*. The approximate location of *oriP*, proximal to *cdc6K* (indicated with arrow), is indicated with a vertical line. The GC skews of the minichromosome and pHH400 are represented by the inner circles.

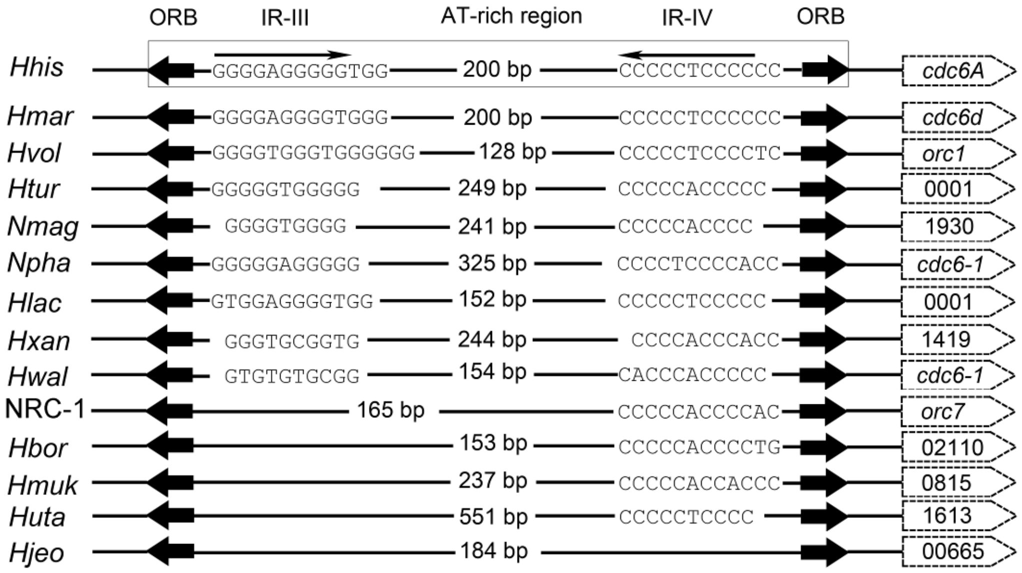

Supplementary Figure S8. The structure (not to scale) of conserved *oriC1* regions in haloarchaea. *Hbor*: *Halogeometricum borinquense*, *Hhis*: *Haloarcula hispanica*, *Hjeo*: *Halalkalicoccus jeotgali* B3, *Hlac*: *Halorubrum lacusprofundi*, *Hmar*: *Haloarcula marismortui*, *Hmuk*: *Halomicrobium mukohataei*, *Htur*: *Haloterrigena turkmenica*, *Huta*: *Halorhabdus utahensis*, *Hvol*: *Haloferax volcanii* DS2, *Hwal*: *Haloquadratum walsbyi*, *Nmag*: *Natrialba magadii*, *Npha*: *Natronomonas pharaonis*, NRC-1: *Halobacterium* sp. NRC-1. The minimal size of *oriC1* in *H. hispanica* is boxed. The inverted ORB elements and the G-rich inverted-repeat (IR-III and IR-IV) are indicated with arrows.

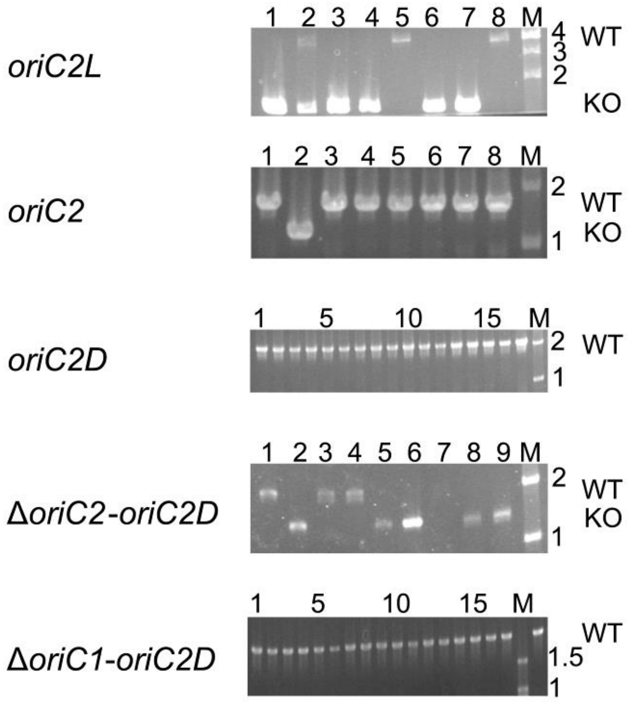

Supplementary Figure S9. PCR assay to screen for knockouts of *oriC2* region. *oriC2L* denotes the whole *oriC2-cdc6E-oriC2D* region. Crude DNA from individual Foa^r colonies was used as template, and forward and backward primers were located ~500 bp up- and down-stream of each target, respectively. Lane M denotes DNA ladder. WT and KO respectively denote wild-type and knockout alleles. The target was considered to be essential after more than 200 Foa^r colonies (including tiny colonies if available) were screened without recovering a single knockout in at least two independent experiments.

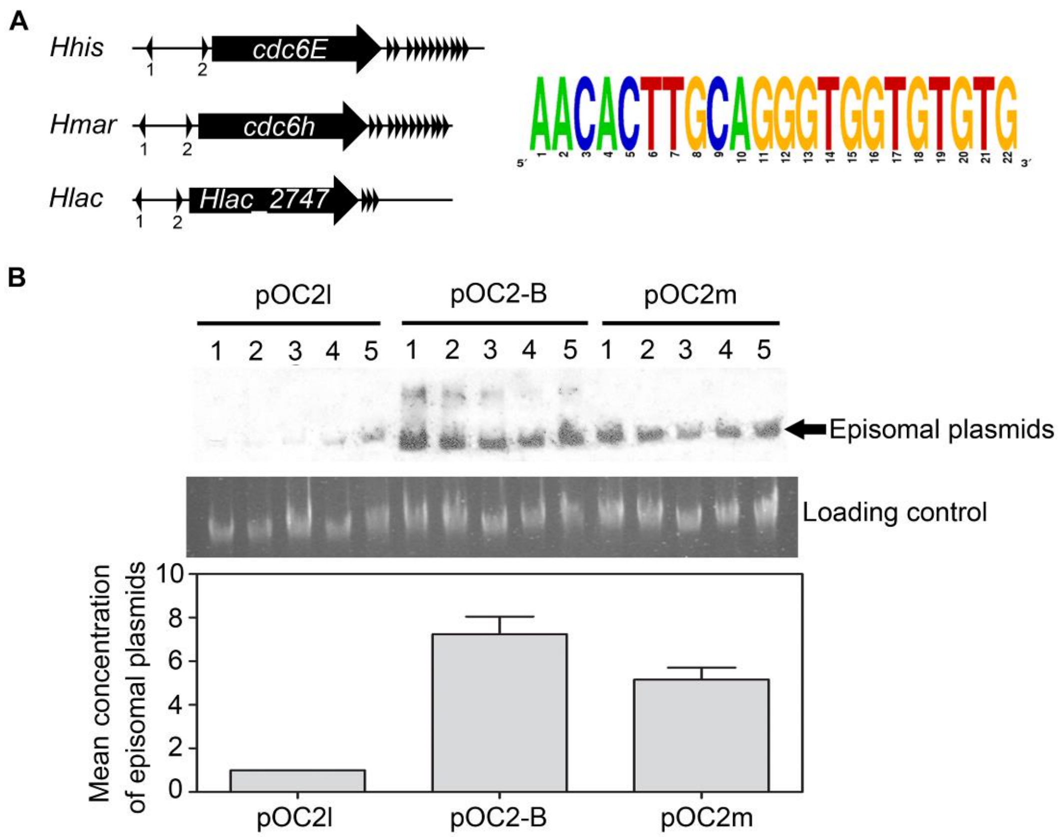

Supplementary Figure S10. Negative control of *oriC2* by the number of ORB elements in the *oriC2D* region. (A) (Left) Physical mapping of *oriC2* homologs in different haloarchaea with *Hhis*, *Hmar* and *Hlac* representing *H. hispanica*, *H. marismortui* and *H. lacusprofundi*, respectively. (Right) Logo representation of ORB elements within the origin region of the three *oriC2* homologs. (B) Southern blot analysis of ARS plasmids in transformants. The three ARS plasmids (pOC2l, pOC2-B and pOC2m) were transformed into the Δ*oriC2L* strain. Five colonies transformed with each ARS plasmid were repeated for Southern blot analysis, and the mean concentration of episomal plasmids was quantified from two independent experiments. The cells were quantified via OD measurements, and genomic DNA was used as a loading control.

Supplemental Table S1. Plasmids and oligonucleotides

**Constructed plasmids and oligonucleotides for knockout experiments**			
**Plasmid**	**Primer name**	**Primer position**	**Sequence (5'-3')***
pHARΔ*cdc6A*	cdc6AFP	5' forward	AAA**CTGCAG**TTACGGTCGCTTTGTCCT
	cdc6AFB	5' reverse	CGC**GGATCC**GCTTTCGTCAGTCATAGTCG
	cdc6ARB	3' forward	CGC**GGATCC**CGACAACTGACCTGCGAACT
	cdc6ARK	3' reverse	CGG**GGTACC**TGTTGCTGTTCGCCGTCA
pHARΔ*cdc6B*	cdc6BFP	5' forward	AAAA**CTGCAG**GAACTCCACTACCACGCC
	cdc6BFB	5' reverse	CGC**GGATCC**TCGTCGCTCATCACTTTCACC
	cdc6BRB	3' forward	CGC**GGATCC**CGAGCAGACAGACCTGGGTT
	cdc6BRK	3' reverse	CGG**GGTACC**TACGCGGCATGGGAGGAT
pHARΔ*cdc6C*	cdc6CFP	5' forward	AAAA**CTGCAG**GCAACGCTGGAGAAGTCG
	cdc6CFB	5' reverse	CGC**GGATCC**GGCTGTACGACCTGCAACA
	cdc6CRB	3' forward	CGC**GGATCC**CGCTTGATTCTCGCTTCG
	cdc6CRK	3' reverse	CGG**GGTACC**CCGTCTGGTCTTCGGCTAC
pHARΔ*cdc6D*	cdc6DFH	5' forward	CCC**AAGCTT**AAGCGAGTTTCCGTGCCA
	cdc6DFP	5' reverse	AAAA**CTGCAG**CTCTATCAAATCCGTCCCT
	cdc6DRP	3' forward	AAAA**CTGCAG**GCATCGACTGCTTCGGGA
	cdc6DRK	3' reverse	CGG**GGTACC**ACATATTCGCGTCTCCCT
pHARΔ*cdc6E*	cdc6EFH	5' forward	CCC**AAGCTT**ACATCCAGCGGAACCTCA
	cdc6EFB	5' reverse	CGC**GGATCC**CCGACGACGAGCAACAGA
	cdc6ERB	3' forward	CGC**GGATCC**TCAGCGGTTGCTTTCGTT
	cdc6ERK	3' reverse	CGG**GGTACC**CGGGCTGTCAGAAAGTAAAG
pHARΔ*cdc6F*	cdc6FFP	5' forward	AAAA**CTGCAG**CGATTTAGACTTGAGGGAT
	cdc6FFB	5' reverse	CGC**GGATCC**AATCAACAACCGCTGCTC
	cdc6FRB	3' forward	CGC**GGATCC**GCTGGGTGAGGGTGAGAACA
	cdc6FRK	3' reverse	CGG**GGTACC**TGTAGATGCCGATGTGGA
pHARΔ*cdc6G*	cdc6GFP	5' forward	AAAA**CTGCAG**GTCCAGGCGGAACTGCTA
	cdc6GFB	5' reverse	CGC**GGATCC**CGGTTAGCGGAGTTGTTTG
	cdc6GRB	3' forward	CGC**GGATCC**CGCACCGTTAATCACAGG
	cdc6GRK	3' reverse	CGG**GGTACC**CCAACGCAAACACGCTAC
pHARΔ*cdc6H*	cdc6HFP	5' forward	AAAA**CTGCAG**GTGAATCGGCAAAGCAGC
	cdc6HFB	5' reverse	CGC**GGATCC**GGATCTGGATTGCCGAAG

	cdc6HRB	3' forward	CGC**GGATCC**GCATGACCGTGAAGAGCA
	cdc6HRK	3' reverse	CGG**GGTACC**TTGAGACTCCACGAGATGC
pHARΔ*cdc6I*	cdc6IFP	5' forward	AAAA**CTGCAG**CAGACCAGTCGGACAAGG
	cdc6IFB	5' reverse	CGC**GGATCC**CGAAGGGAACTAACACGAAG
	cdc6IRB	3' forward	CGC**GGATCC**TCTGAACGCCCTCCTGAA
	cdc6IRK	3' reverse	CGG**GGTACC**TCTTCTCGTGGGTTCTCC
pHARΔ*cdc6J*	cdc6JFP	5' forward	AAAA**CTGCAG**CTGTCGGCGTAAGTTTGG
	cdc6JFB	5' reverse	CGC**GGATCC**CGCTGTTCGGTTGGGTAT
	cdc6JRB	3' forward	CGC**GGATCC**GCGGTGGAAAGTACAAGGAA
	cdc6JRK	3' reverse	CGG**GGTACC**CGTAGTAGTCGCTGGTGAGAAA
pHARΔ*cdc6K*	cdc6KFP	5' forward	AAAA**CTGCAG**AGTCGGGAACAGAACCAT
	cdc6KFB	5' reverse	CGC**GGATCC**ACTAACCTCGTTGATAGCG
	cdc6KRB	3' forward	CGC**GGATCC**CCCCATAGTCGTTGCCTGAT
	cdc6KRK	3' reverse	CGG**GGTACC**TAGGCGAGGAATGGGAGA
pHARΔ*oric1*	oric1FH	5' forward	CCC**AAGCTT**CTCGACGTTCTCCTTTCG
	oric1FP	5' reverse	AAA**CTGCAG**ATCCGAATCGCTGGGT
	oric1RP	3' forward	AAA**CTGCAG**CCGTCTAACCCAGTGAA
	oric1RK	3' reverse	CGG**GGTACC**TTTGTGCGGCGTGTAG
pHAR Δ*oric1-cdc6A*	OC1FH	5' forward	CCC**AAGCTT**TCTCCATGTCGTGTTCGAGG
	OC1FP	5' reverse	AAAA**CTGCAG**CCCAGGGATGTCTGTCGTT
	OC1RP	3' forward	AAAA**CTGCAG**CGACAACTGACCTGCGAACT
	OC1RK	3' reverse	as same as cdc6ARK
pHARΔ*oric2U*	oric2FH	5' forward	CCC**AAGCTT**TCCGCTGGAATGTTGT
	oric2FB	5' reverse	CGC**GGATCC**ACGGTGGTCACTGGTTTT
	oric2RB	3' forward	CGC**GGATCC**CCATAGCGAACGGTTGGTC
	oric2RK	3' reverse	CGG**GGTACC**GGACGATCTCCCAGTTCAGG
pHAR Δ*oric2-cdc6E*	OC2FH	5' forward	as same as cdc6EFH
	OC2FB	5' reverse	as same as cdc6EFB
	OC2RB	3' forward	CGC**GGATCC**CCATAGCGAACGGTTGGTC
	OC2RK	3' reverse	CGG**GGTACC**GGACGATCTCCCAGTTCAGG
pHARΔ*oric2L*	OC2LFH	5' forward	CCC**AAGCTT**GGTGACGCTGTACGCCAAGA
	OC2LFB	5' reverse	CGC**GGATCC**ACTGAGCCCTCTCCGTTACAT
	OC2LRB	3' forward	as same as OC2RB

	OC2LRK	3' reverse	as same as OC2RK
pHARΔ*oric2D*	OC2DFH	5' forward	as same as OC2LFH
	OC2DFB	5' reverse	as same as OC2LFB
	OC2DRB	3' forward	CGC**GGATCC**CAAATACGCTGGCTACG
	OC2DRK	3' reverse	CGG**GGTACC**TGCTGGAACCCAAAGTG
pHAR	OC3FH	5' forward	CCC**AAGCTT**TCATTCTTGCTCGTTGGGT
Δ*oric3-cdc6D*	OC3FP	5' reverse	AAAA**CTGCAG**TAAGCCCCGTTAAACGTGTT
	OC3RP	3' forward	as same as cdc6DRP
	OC3RK	3' reverse	as same as cdc6DRK
pHARΔ*oric4*	oriC4FP	5' forward	AAAA**CTGCAG**CCCCAACCAGTGTATC
	oriC4FB	5' reverse	CGC**GGATCC**GTCTGACGCTTCTTGC
	oriC4RB	3' forward	CGC**GGATCC**ATCATGTAGGCGATTGAGCT
	oriC4RK	3' reverse	CGG**GGTACC**AGCTGTCCGAAGTTCATCTC
pHAR	OC4FP	5' forward	as same as cdc6GFP
Δ*oric4-cdc6G*	OC4FB	5' reverse	as same as cdc6GFB
	OC4RB	3' forward	as same as oriC4RB
	OC4RK	3' reverse	as same as oriC4RK
pHARΔ*oric5*	oriC5FP	5' forward	AAAA**CTGCAG**GCGAGACGAAGACAGTATGA
	oriC5FB	5' reverse	CGC**GGATCC**CATTCAGAGTCGGAAGTCAT
	oriC5RB	3' forward	CGC**GGATCC**GATGGGAACTGAGCGACTG
	oriC5RK	3' reverse	CGG**GGTACC**CGGTAATAGTCCGAGGT
pHAR	OC5FP	5' forward	as same as oriC5FP
Δ*oric5-cdc6H*	OC5FB	5' reverse	as same as oriC5FB
	OC5RB	3' forward	as same as cdc6HRB
	OC5RK	3' reverse	as same as cdc6HRK
pHARΔ*oric6*	oriC6FH	5' forward	CCC**AAGCTT**CTGGTCAGTCGA
	oriC6FB	5' reverse	CGC**GGATCC**ATCTACTACCCTCGCCTA
	oriC6RB	3' forward	CGC**GGATCC**GTGAGCCTCAAAATAACC
	oriC6RK	3' reverse	CGG**GGTACC**CAAAGTGCCTACCAGA
pHAR	OC6FP	5' forward	as same as cdc6IFP
Δ*oric6-cdc6I*	OC6FB	5' reverse	as same as cdc6IFB
	OC6RB	3' forward	CGC**GGATCC**CGGATTTCTGGTGTTTCG
	OC6RK	3' reverse	CGG**GGTACC**CTGGCTGAGTCTGAGCATAT

pHARΔ*oric7*	oriC7FH	5' forward	AAAA**CTGCAG**ATGCCAGCGGAGACAGCA
	oriC7FB	5' reverse	CGC**GGATCC**CCAGTTTCGCTCGCACCA
	oriC7RB	3' forward	CGC**GGATCC**TATACCCAACCGAACAGC
	oriC7RK	3' reverse	CGG**GGTACC**TTCCGTTACGAGTTCATC
pHAR	OC7FP	5' forward	as same as oriC7FH
Δ*oric7-cdc6J*	OC7FB	5' reverse	as same as oriC7FB
	OC7RB	3' forward	as same as cdc6JRB
	OC7RK	3' reverse	as same as cdc6JRK
pHARΔ*oriP*	oriPFP	5' forward	AAAA**CTGCAG**CGCTGAACTTAGGTTGTC
	oriPFB	5' reverse	CGC**GGATCC**AGGGTCCCGCCATTCTTT
	oriPRB	3' forward	CGC**GGATCC**GCTGTTCCCTTCCATCTTG
	oriPRK	3' reverse	CGG**GGTACC**AAGCGACGCAATCAAACG
pHAR	OPFP	5' forward	as same as cdc6KFP
Δ*oriP-cdc6K*	OPFB	5' reverse	as same as cdc6KFB
	OPRB	3' forward	as same as oriPRB
	OPRK	3' reverse	as same as oriPRK

**Constructed plasmids and oligonucleotides for gene disruption experiments**		
**Plasmid**	**Primer name**	**Sequence (5'-3')***
pUBP-D*cdc6A*	Dcdc6AFB	CGC**GGATCC**TCTATGGCAAGACTGGAACG
	Dcdc6ARK	CGG**GGTACC**TGGACTTGACCCGTGGAT
pUBP-D*cdc6I*	Dcdc6IFB	CGC**GGATCC**CGATTTCACCCGCCTTTC
	Dcdc6IRK	CGG**GGTACC**CTGGACGACATTATCACCTACC
pUBP-D*cdc6K*	Dcdc6KFB	CGC**GGATCC**GATCTGGATCAGCTCCTTCG
	Dcdc6KRK	CGG**GGTACC**CGGGCGTGACACTGAACTA

**Constructed plasmids and oligonucleotides for ARS assays**		
**Plasmid**	**Primer name**	**Sequence (5'-3')***
pOC1-A	ARSoriC1FP	AAAA**CTGCAG**AATCCCATCCTATCCTCC
	ARSoriC1RH	CCC**AAGCTT**CGCTTTCGTCAGTCATAGT
pOC1-B	ARSOC1FP	as same as ARSoriC1FP
	ARSOC1RH	CCC**AAGCTT**TAGCCGTATCACTCGAAG
pOC2-A	ARSoriC2FN	CATG**CCATGG**CAGCGGTTGCTTTCGT
	ARSoriC2FB	CGC**GGATCC**ACGGTGTCTACGGGCTG
pOC2-B	ARSOC2FN	CATG**CCATGG**CTACGTGAACTGCCAGAGG

	ARSOC2RB	as same as ARSoriC2FB
pOC2l	ARSOC2lFN	CATG**CCATGG**CGGAGAGGGCTCAGTCG
	ARSOC2lRB	as same as ARSoriC2FB
pOC2m	ARSOC2mFN	CATG**CCATGG**TCACGGTCTACTGTTGGC
	ARSOC2mRB	as same as ARSoriC2FB
pOC2d	ARSOC2dFN	as same as ARSoriC2FN
	ARSOC2dRB	CGC**GGATCC**TTCCAGAGGGATCGAAGA
pOC6-A	ARSoriC6FN	CATG**CCATGG**TGAACGCCCTCCTGAA
	ARSoriC6RB	CGC**GGATCC**GTTCGGAGTGGGTTGTTG
pOC6-B	ARSOC6FN	CATG**CCATGG**ACGGAGTCGAGGTAGGG
	ARSOC6RB	as same as ARSoriC6RB
pOC7-A	ARSoriC7FB	CGC**GGATCC**GTGAGTGATCGGGAGCC
	ARSoriC7RB	CGC**GGATCC**TGGAGGTGGGCGAAAAC
pOC7-B	ARSOC7FB	as same as ARSoriC7FB
	ARSOC7RB	CGC**GGATCC**CATCGTAAGCAGCGGTT
pOP-A	ARSoriPFN	CATG**CCATGG**CCATAGTCGTTGCCTGAT
	ARSoriPRB	CGC**GGATCC**TAGCGTAGAAGGTATCGATGC
pOP-B	ARSOPFN	CATG**CCATGG**CCATAGTCGTTGCCTGAT
	ARSOPRB	as same as ARSoriPRB

Oligonucleotides for real-time PCR and truncation and mutation analyses of *oriC1* origin

**Primer**	**Sequence (5'–3')***	**Relevant properties**
oriC1SF	GACTGACGAAAGCGACAACTC	Forward primer for synthesis of the real-time PCR standard for *oriC1* region
oriC1SR	TGGACTTGACCCGTGGAT	Reverse primer for synthesis of the real-time PCR standard for *oriC1* region
oriC1DF	CTCATCTATGGCAAGACT	Forward primer for detection of the DNA copies in *oriC1* region
oriC1DR	GTTGATGTACTCCACTTC	Reverse primer for detection of the DNA copies in *oriC1* region
oriC2SF	TTACGAAGTGGGTACGGGACA	Forward primer for synthesis of the real-time PCR standard for *oriC2* region
oriC2SR	TCGCTGTAGGACGACTGGA	Reverse primer for synthesis of the real-time PCR standard for *oriC2* region
oriC2DF	GAACTGCTGGATTACTTC	Forward primer for detection of the DNA copies in *oriC2* region

oriC2DR	GAGGAGTAGAGGTTGTAA	Reverse primer for detection of the DNA copies in *oriC2* region
oriC1mF	AAAA**CTGCAG**TAGGGGTGGACCATCCT	Primers for truncation analysis of *oriC1* origin
oriC1mR	CCC**AAGCTT**TAGGACAAAGCGACCGT	
D1F	AAAA**CTGCAG**AACGACAGACATCCCT	
D2R	CCC**AAGCTT**AACGGGAAACAGGCC	
SMIF	GGTGGACCATCCTAG*TTTTTT*ACCCCTTTGTTTCAGG	Primers for mutation at the I region in *oriC1* origin
SMIR	CCTGAAACAAAGGGGT*AAAAAA*CTAGGATGGTCCACC	
SMIIF	GGAAACGGAGGGGT*AAAAAAAA*TTGGCCCGTTACGGT	Primers for mutation at the II region in *oriC1* origin
SMIIR	ACCGTAACGGGCCAA*TTTTTTTT*ACCCCTCCGTTTCC	
SMIIIF	AACGACAGACATCCCT*AAAAGAAAAA*TGGAGAATTACCGAT C	Primers for mutation at the III region in *oriC1* origin
SMIIIR	GATCGGTAATTCTCCA*TTTTTCTTTT*AGGGATGTCTGTCGTT	
SMIVF	ATTTTCGTCACCCTCA*TTTTTCTTTT*CCGGGCCTGTTTGG	Primers for mutation at the IV region in *oriC1* origin
SMIVR	CCAAACAGGCCCGG*AAAAGAAAAA*TGAGGGTGACGAAAAT	

* Sequences representing restriction and mutation sites are highlighted in bold and italic, respectively.

Supplemental Table S2. Differential gene expression of *cdc6* genes (exponential vs. stationary phase) from transcriptome analyses.

**Replicon**	**Gene**	**Fold change of gene expression (Exponential/stationary)***	***cdc6*-associated origin**
Chromosome	***cdc6A***	1.6639 ±0.4541	***oriC1-cdc6A***
	cdc6B	1.8026 ±0.1815	
	cdc6C	-	
	cdc6D	-	
	cdc6E	1.5083 ±0.4051	***oriC2-cdc6E***
	cdc6F	1.0372 ±0.0899	
Minichromosome	*cdc6G*	-	*oriC4-cdc6G*
	cdc6H	0.6996 ±0.2055	*oriC5-cdc6H*
	cdc6I	2.2140 ±0.6160	***oriC6-cdc6I***
	cdc6J	1.4135 ±0.1371	***oriC7-cdc6J***
pHH400	***cdc6K***	0.5818 ±0.1942	***oriP-cdc6K***
	polB1	3.0707 ±0.3625	
	polA1	1.7667 ±0.3590	
	polA2	3.6475 ±0.5850	

* DNA polymerase genes (*polB1*, *polA1* and *polA2*) are provided as positive control of the transcriptome results. Five functional replication origins identified via ARS activity are bolded.
